# Nano-/Microrobots for Environmental Remediation in the Eyes of Nanoarchitectonics: Toward Engineering on a Single-Atomic Scale

**DOI:** 10.34133/research.0624

**Published:** 2025-02-24

**Authors:** Anna Jancik-Prochazkova, Katsuhiko Ariga

**Affiliations:** ^1^ Research Center for Materials Nanoarchitectonics, National Institute for Materials Science (NIMS), Tsukuba 305-0044, Japan.; ^2^ Graduate School of Frontier Sciences, The University of Tokyo, Kashiwa 277-8561, Japan.

## Abstract

Nano-/microrobots have been demonstrated as an efficient solution for environmental remediation. Their strength lies in their propulsion abilities that allow active “on-the-fly” operation, such as pollutant detection, capture, transport, degradation, and disruption. Another advantage is their versatility, which allows the engineering of highly functional solutions for a specific application. However, the latter advantage can bring complexity to applications; versatility in dimensionality, morphology, materials, surface decorations, and other modifications has a crucial effect on the resulting propulsion abilities, compatibility with the environment, and overall functionality. Synergy between morphology, materials, and surface decorations and its projection to the overall functionality is the object of nanoarchitectonics. Here, we scrutinize the engineering of nano-/microrobots with the eyes of nanoarchitectonics: we list general concepts that help to assess the synergy and limitations of individual procedures in the fabrication processes and their projection to the operation at the macroscale. The nanoarchitectonics of nano-/microrobots is approached from microscopic level, focusing on the dimensionality and morphology, through the nanoscopic level, evaluating the influence of the decoration with nanoparticles and quantum dots, and moving to the decorations on molecular and single-atomic level to allow very fine tuning of the resulting functionality. The presented review aims to lay general concepts and provide an overview of the engineering of functional advanced nano-/microrobot for environmental remediation procedures and beyond.

## Introduction

The environmental scenarios of plastic waste occupying the deep oceans, waste flying out in space, and water scarcity have become reality [1–3]. Although there are already established methods for waste reduction, management, and recycling, they do not fully reflect the actual situation and needs [4–9]. The aforementioned scenarios are just the visible tip of the iceberg; low-dimensional, molecular, and atomic pollutants such as nano-/microplastics, drug residues, heavy metals, oil contaminants, and various organic substances occupy the environment and enter and directly influence the bodies of living organisms via the food chain [10,11]. In particular, the accumulation of nano-/microplastics is a burning environmental issue; they are commonly found in living organisms, including humans. There are still gaps in their detection, quantification, and removal procedures [12,13]. Similarly, polyfluorinated alkyl substances represent abundant contamination with cumulative capabilities [14]. Humankind and governments are fully aware of the current risks related to the accumulation and circulation of harmful waste and are taking relevant actions to limit its excessive purposeless use and promote recycling [15]. Contributions to environmental remediation have also been brought about by the scientific community, which is fully aware of the importance of this situation. A recent novel approach to environmental remediation is the use of nano-/microrobots—smart, universal, and efficient tiny machines that can contribute to the detection, capture, degradation of pollutants, and beyond [16,17].

Nano-/microrobots are multifunctional devices designed to be autonomously propelled in a defined environment to achieve a specific task [18]. As they are autonomously driven by chemical and physical principles, their fabrication does not involve any elaborate electronic approaches or software programming; it ensures versatility and easy scalability of their application and cost efficiency while reaching the required precision. Depending on the application requirements, nano-/microrobots can be programmed to move, capture, detect, transport, degrade, catalyze, and communicate, among others. In combination with their propulsion abilities, they can actively perform the desired task in the so-called “on-the-fly” mode, which dramatically enhances their efficiency compared to static systems [19–21]. Based on the scope of the skills listed, they can be used in bioapplications, medical applications, environmental applications, the food and chemical industry, and others [22]. Programming is achieved through the choice of material or their combination, the morphology design, and effective functionalization. In principle, there is no universal nano-/microrobot; specific design and functionality must be tailored for a specific task or for a set of tasks of a similar concept [23]. There are several universal approaches to the engineering of nano-/microrobots—the general set of approaches arises through different scientific fields, such as computation, organic/inorganic and supramolecular chemistry, nanotechnology, physical chemistry, physics, and statistics [24]. As the multidisciplinary character is more than palpable, nanoengineering of highly functional materials might appear very specific. This is where nanoarchitectonics finds its place as a separate scientific field that postulates and unifies general concepts for the fabrication of advanced dynamic functional materials arising from the nanoscale [25].

Nanoarchitectonics was first postulated at the beginning of the 21st century by Masakazu Aono and has been experiencing a great boom since then. It is defined as a set of principles that allows the precise fabrication of functional smart nanosystems from individual atomic units, molecular units, and nano-units [26]. It not only defines the interactions between individual units but also predicts and explains the resulting properties of functional dynamic systems at the macroscopic scale [27]. As already noted, nanoarchitectonics overtook its approaches and mechanisms in multiple fields, such as general chemistry, organic, supramolecular and physical chemistry, physics, biology, statistics, and others, thus providing complex concepts and tools toward engineering, fabrication, and characterization of dynamic functional systems with control at the atomic scale [28,29].

**Fig. 1.**
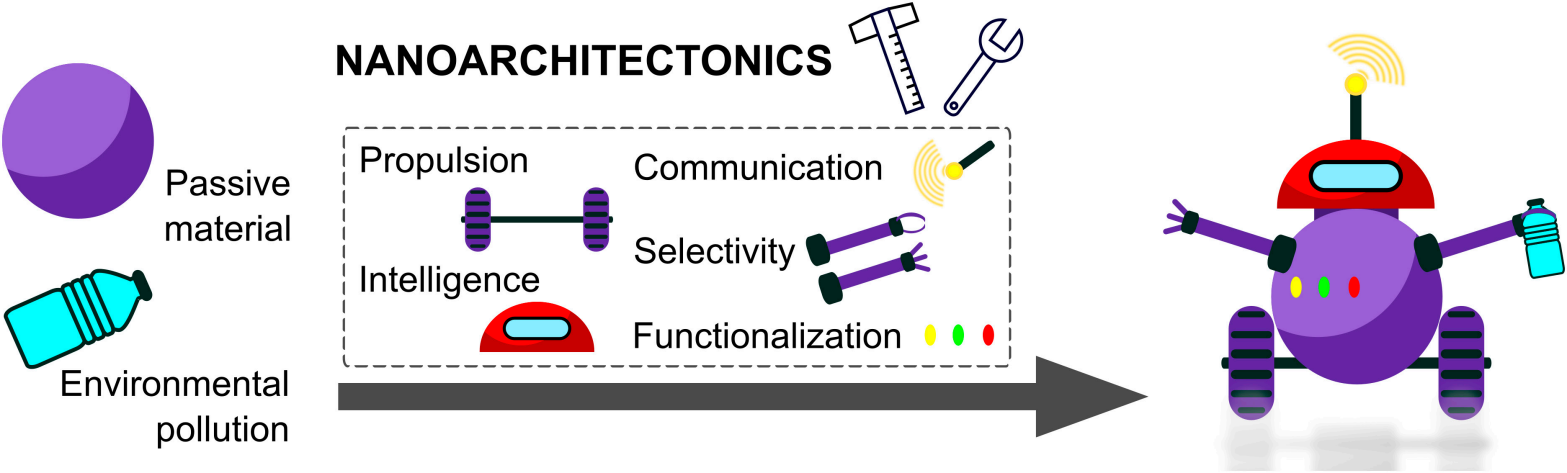
Nanoarchitectonics as a tool for fabrication of nano-/microrobots.

With the attention paid to nanoarchitectonics, the engineering of advanced functional nano-/microrobots is straightforward. It aims to tune the design and morphology of nano-/microrobots for desired applications via a defined set of concepts. Exaggeratedly, nanoarchitectonics can be understood as the LEGO puzzle; it provides universal building blocks that can be arbitrarily combined to fabricate the desired dynamic designs with different morphologies and functionalities (Fig. 1). With this in mind, the presented review summarizes the general approaches toward the engineering of nano-/microrobots in the eyes of nanoarchitectonics to provide an overview of the available concepts toward the fabrication of highly efficient dynamic nano-/microrobotic systems. Unlike in comprehensive similarly oriented reviews in the field of nano-/microrobotics applicable in environmental applications [30–32], this work aims to focus on the aspect of nanoengineering. Using the tools of nanoarchitectonics, the approaches and mechanisms of nano-/microrobot fabrication and their functionalization are discussed with respect to descending dimensionality. Providing the latest trends in the methodology of nanoarchitectonics, this review aims to become a complex guide for engineering of nano-/microrobots beyond the environmental remediation.

## Nanoarchitectonics as a General Concept for the Fabrication of Dynamic Nanosystems

Nanoarchitectonics is an established methodology for the precise nanoengineering of dynamic and functional materials. It was proposed in 2000 to fill conceptual gaps in the field of nanotechnologies [33]. At the nanoscale, certain specific phenomena, such as thermal/statistical fluctuations, mutual interactions, and quantum effects, cannot be overlooked because they dramatically influence the resulting structures and their properties at the macroscale. Where nanotechnology provides technologies for the observation, analysis, and manipulation of nanoscale objects, nanoarchitectonics aims to exploit nanoscale objects, organize them into advanced functional dynamic systems, and assess the synergy between functionalities and their projection into macroscale [26]. Nanoarchitectonics is a highly multidisciplinary field that collects knowledge and approaches from nanotechnologies, organic and inorganic chemistry, supramolecular chemistry, physical chemistry, colloidal chemistry, computation chemistry, and others. Consequently, nanoarchitectonics provides a set of tools for atomic/molecular manipulation, material creation, and advanced device fabrication [34].

To provide a clearer idea about nanoarchitectonics and the results of its concepts, a limited overview of dynamic material systems could be atomic switches, molecular machines and rotors, self-assembled monolayers, layer-by-layer (LBL) assemblies, Langmuir–Blodgett films, and others. Nanoarchitectonics also finds great inspiration from biorelated fields, such as DNA machines, DNA origami, and various bilayer systems mimicking cell membranes. The synergy arising from the nanoscale can lead to the engineering of highly ordered systems, providing a solution for molecular manipulation, switchable catalysis, self-healing abilities, light-harvesting properties, and molecular recognition [35]. As a result, complex and smart systems operating macroscopically, such as neuromorphic networks, environmental applications, functional electronic devices, and life science, can be designed by implementing the principles and approaches of nanoarchitectonics [36].

## Introduction to Nano-/Microrobots

**Fig. 2.**
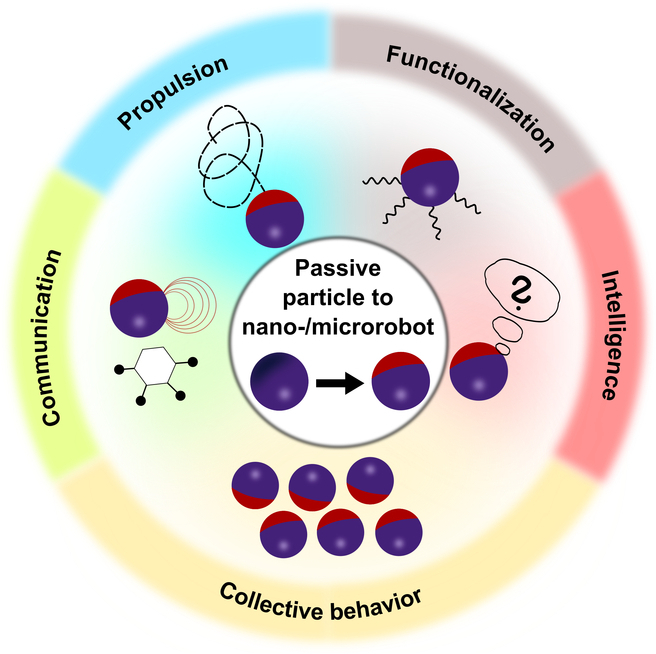
Schematic illustration of different aspects in nanoarchitecting nano-/microrobots from passive particles.

Nano-/microrobots are artificial autonomous systems designed to actively accomplish a desired task. To design a functional and smart nano-/microrobot, 2 important aspects must be considered to differentiate nano-/microrobots from conventional static nano-/microparticles: propulsion abilities and advanced functionality (Fig. 2) [37]. The functionality can differ substantially depending on the requirements of the desired application: nano-/microrobots can be provided the ability to capture, collect, transfer, release, degrade, etc.; they can be designed to exhibit a specific collective behavior and taxis; they can communicate with each other or with the environment and sense; eventually, they can be programmed with a certain intelligence [38]. The individual abilities and functionalities are summarized below to provide a general overview of the concepts of nano-/microrobotics. Specific approaches in the nanoarchitectonics of nano-/microrobots are discussed separately in the “Nanoarchitectonics of Nano-/Microrobots for Environmental Remediation” section.

### Propulsion abilities

Propulsion abilities are key features of nano-/microrobots. There are many different approaches to the propelling of nano-/microrobots; in general, they can be classified as (a) chemically fueled, where the decomposition of the fuel generates chemical gradients that induce locomotion [39], and (b) fueled by an external field, such as light irradiation, magnetic field, and electric or acoustic field [40]. The choice of propulsion mechanism depends on the requirements and limitations of a specific application. For example, in medical applications, the compatibility of nano-/microrobots is limited when hydrogen peroxide (H_2_O_2_) fuel and/or ultraviolet (UV) irradiation are used to induce propulsion [41]. In contrast, in the case of biofilm eradication, it is highly advantageous to use the synergy of propelled nano-/microrobots together with the slightly toxic effect of the H_2_O_2_ fuel and/or UV irradiation [42]. Notably, propulsion modes can be combined [43]. The use of magnetic navigation is very convenient to be combined with fuel-driven propulsion abilities as the latter enables task accomplishment in the “on-the-fly” mode, whereas magnetic abilities allow precise navigation and potential regeneration [44].

Finally, depending on the requirements, choice of the material, design, and source of propulsion abilities, nano-/microrobots can be provided with collective behavior to act as one body rather than multiple autonomous objects. From this perspective, nano-/microrobots can be designed to exhibit swarming abilities, schooling behavior, self-assembly, and self-orientation properties [45].

In the following, different propulsion modes and their respective mechanisms are briefly discussed.

#### Propulsion of nano-/microrobots by chemical fuels

**Fig. 3.**
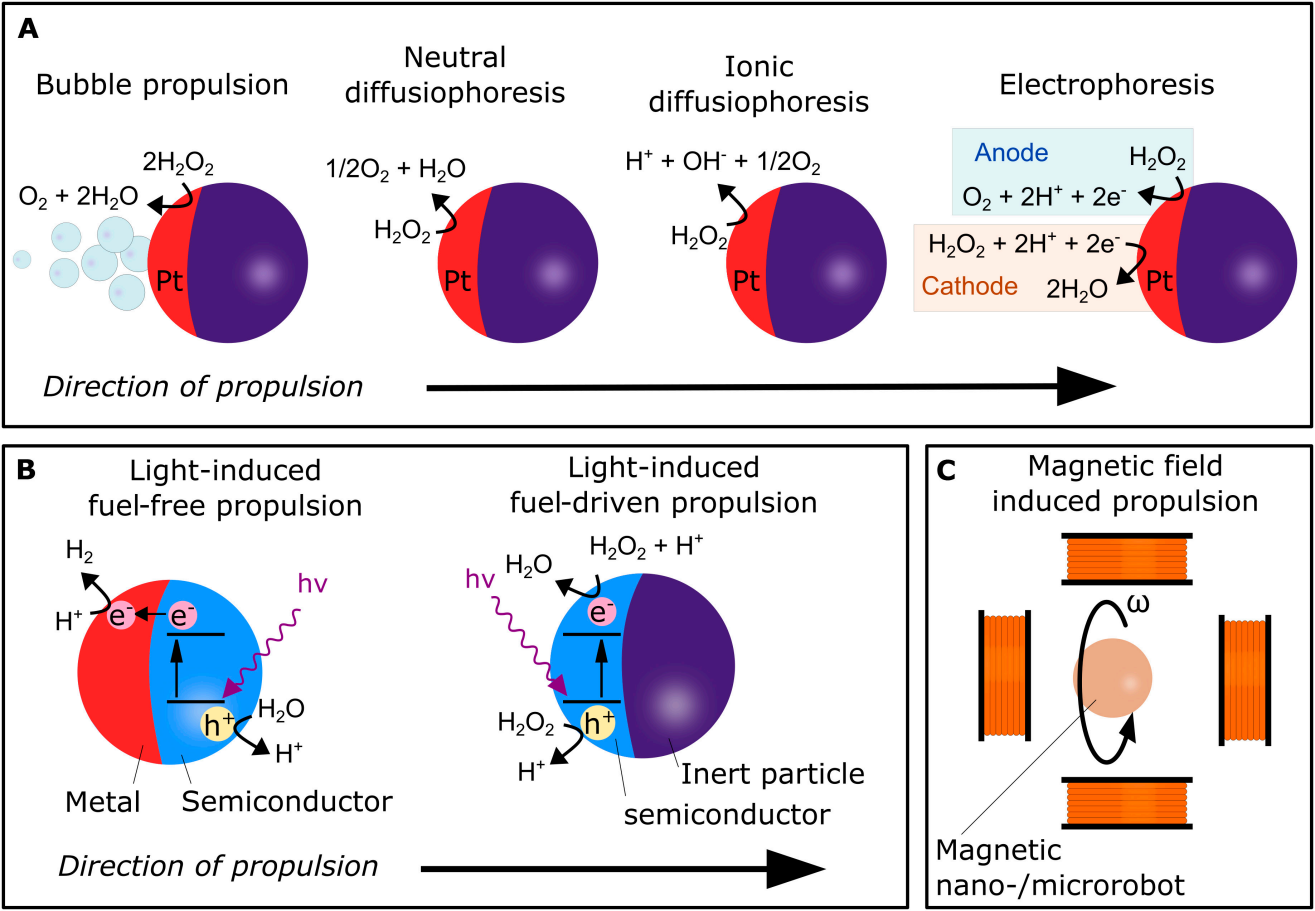
Illustration of selected propulsion mechanisms. (A) Chemically powered Janus-type nano-/microrobots and mechanisms of H_2_O_2_ decomposition over platinum moiety. (B) Light-induced propulsion and its mechanisms in fuel-free aqueous environment and fueled with H_2_O_2_. (C) Propulsion of nano-/microrobots in a rotating magnetic field of an applied frequency ω.

Fuels are chemical substances that decompose over the body of nano-/microrobots upon the creation of chemical gradients. If the gradients are formed nonuniformly around the surface of nano-/microrobots, they can become the driving force for propulsion. In the most traditional approach, H_2_O_2_ is a common fuel that catalytically decomposes over the body of nano-/microrobots (Fig. 3A). To ensure nonhomogeneous formation of chemical gradients, Janus-type nano-/microrobots are often designed. Janus nano-/microrobots comprise an inactive core and catalytically active partial surface coating that can be achieved by a nonhomogeneous platinum coating or eventually by decoration with enzyme peroxidase [46]. In both cases, H_2_O_2_ decomposes into water and oxygen according to Eq. 1.

$${\text{2H}}_{2}O_{2}\to {\text{2H}}_{2}O+O_{2}$$ (1)

Oxygen, as a product of the decomposition of H_2_O_2_, induces an uneven chemical gradient around the catalytically active moiety of the nano-/microrobots, which is the driving force for propulsion. Although H_2_O_2_ decomposition can be described by a straightforward reaction scheme in Eq. 1, more in-depth propulsion mechanisms are still not fully understood. At a certain concentration of oxygen, nano-/microrobots can be efficiently propelled by the formation of oxygen bubbles. The formation of bubbles is observed by the naked eye when the reaction is vigorous, typically in an excessive concentration of H_2_O_2_. However, this concept requires high concentrations of H_2_O_2_ exceeding 3 wt %, which can be considered a potential hazard in certain applications [47]. The use of a lower H_2_O_2_ concentration can still lead to an efficient propulsion that allows “on-the-fly” achievement of tasks. In this case, the mechanism can be explained as neutral self-diffusiophoresis, where nonhomogeneously generated O_2_ molecules induce a chemical gradient. However, it is believed that propulsion does not arise purely from the formation of an oxygen gradient, rather than from the generation of charged intermediate species that are formed during the decomposition of H_2_O_2_ according to Eq. 2:

$$H_{2}O_{2}\to H^{+}+{\text{OH}}^{-}+1\;/\;2O_{2}$$ (2)

Because the ionic gradient induces propulsion, this mechanism is referred to as ionic diffusiophoresis [48,49].

Different suggested fuel-driven propulsion mechanisms assume that the thickness of the platinum layer in Janus-type nano-/microrobots is not homogeneous along the metallic cap; therefore, the area of the thinner coating can act as an anode that decomposes H_2_O_2_ into protons, oxygen, and electrons, as suggested in Eq. 3. The conductive properties of platinum allow charge transfer to regions with a thicker platinum coating that can be considered as a cathode and can allow the reaction of H_2_O_2_ with protons and electrons upon the generation of H_2_O, as shown in Eq. 4.

$$\text{Anode}:H_{2}O_{2}\to {\text{2H}}^{+}+O_{2}+{\text{2e}}^{–}$$ (3)

$$\text{Cathode}:H_{2}O_{2}+{\text{2H}}^{+}+{\text{2e}}^{-}\to {\text{2H}}_{2}O$$ (4)

The latter mechanism behind fuel-induced propulsion abilities should be referred to as self-electrophoresis, as electric field generation is the key driving force of propulsion [50].

As noted above, the controversial use of H_2_O_2_ as a chemical fuel in the field of nano-/microrobotics has encouraged scientists to look for alternative fuels, especially in combination with the selective decomposition over enzymes attached to the main body of nano-/microrobots [51]. For example, nano-/microrobots propelled by the decomposition of urea over enzyme urease have been studied [52]. Alternatively, glucose can be used to propel nano-/microrobots by decorating the surface of nano-/microrobots with a suitable enzymatic system [53]. Different concepts in which nano-/microrobots can be fuel carriers have also been developed. In this configuration, magnesium-based nano-/microrobots exhibit propulsion in aqueous environments owing to the reaction of Mg with water, resulting in the evolution of the H_2_ bubbles [54]. Similarly, AgCl-based nano-/microrobots decompose in an aqueous environment under UV light exposure, releasing Ag^+^ and Cl^−^ ions that generate an electrochemical gradient in the surrounding environment [55].

Finally, it is worth noting that the use of fuel-driven nano-/microrobots can encounter several limitations in real-scale applications, particularly when employing diffusiophoresis and electrophoresis propulsion mechanisms. Phoretic propulsion mechanisms are enabled by the generation of ionic species, which can be hindered in systems containing additional chemical substances and ionic species. Therefore, the use of fuel-driven nano-/microrobots is limited in complex aqueous media such as seawater or biological fluids. Another significant disadvantage is impossibility of the remote motion control and the possibility of complete fuel consumption or functional surface eradication, resulting in the loss of propulsion ability [56].

#### External field-induced propulsion

The use of external fields, such as light irradiation, magnetic field, ultrasound, and electric field, among others, to induce the propulsion of nano-/microrobots seems more convenient because of the absence of fuels that may not be compatible with the desired system [57]. The use of an external field can also provide the possibility of direct control of propulsion by simply starting or stopping the source of external field. Eventually, precise navigation of nano-/microrobots can be achieved by changing the external field source orientation. The limitations associated with fuel consumption and nano-/microrobot eradication are also forestalled. However, the use of each external field can encounter several limitations, such as the complexity of the setup or limited permeation through the medium of operation [58,59].

##### Light-driven nano-/microrobots

Light-driven nano-/microrobots are typically engineered by selecting semiconductor-based photocatalytic materials. Commonly employed light-activated materials include TiO_2_, ZnO, BiVO_4_, hematite, and organic semiconductors [60]. The mechanism of light-induced propulsion originates in light absorption and the formation of electrons and holes, which interact with the environment and generate ionic species. As the generated gradients are traditionally insufficient to propel nano-/microrobots, 2 additional approaches can be targeted (Fig. 3B). First, the electronic properties of the material can be tuned by decorating the surface with a metallic layer or a different (semi)conductive material to achieve efficient charge separation, thereby increasing the generation of ionic species upon reaction with the environment according to Eqs. 5 and 6 [61]. In this approach, local electric current is generated by transporting electrons to the metallic moiety where they react with protons. This mechanism is referred to as self-electrophoresis.

$${\text{2H}}_{2}O+{\text{4h}}^{+}\to O_{2}+{\text{4H}}^{+}$$ (5)

$${\text{4H}}^{+}+{\text{4e}}^{-}\to {\text{2H}}_{2}$$ (6)

Second, propulsion can be achieved in the presence of H_2_O_2_, which acts as a fuel that photocatalytically decomposes over the semiconductive material according to Eqs. 7 and 8 inducing propulsion via self-diffusiophoresis.

$$H_{2}O_{2}+{\text{2h}}^{+}\to O_{2}+{\text{2H}}^{+}$$ (7)

$$H_{2}O_{2}+{\text{2e}}^{-}+{\text{2H}}^{+}\to {\text{2H}}_{2}O$$ (8)

In most cases, both approaches are combined to provide nano-/microrobots with efficient propulsion. In particular, the photocatalytic decomposition of H_2_O_2_ typically requires lower fuel concentrations than pure fuel-driven nano-/microrobots (even below 0.1 wt %) [49,62].

UV light is typically applied to induce propulsion of light-active nano-/microrobots, which represents several limitations. First, UV light can be considered harmful, particularly when combined with biorelated applications. Second, UV light cannot permeate tissues. Third, UV light represents only a part of the entire spectrum, potentially limiting the powerful source of daylight. From this point of view, it is important to seek materials that can provide nano-/microrobots with the ability to be propelled under visible light and eventually under near-infrared (NIR) light. The use of visible or NIR light also allows the use of different propulsion mechanisms, such as photothermal effects arising from the formation of the temperature gradient around the body of nano-/microrobots [63].

##### Magnetic field-driven nano-/microrobots

The nano-/microrobot fabrication can be approached by incorporating magnetic materials that allow propulsion and navigation in a magnetic field [64]. Figure 3C illustrates an example of the electromagnetic actuation system using multiaxial Helmholtz coils that can efficiently navigate nano-/microrobots. The resulting trajectories and speed can be efficiently controlled and regulated depending on the morphology and applied frequency. In particular, the morphology is projected in the mechanism of propulsion; magnetic rollers, wigglers, surface walkers, corkscrew-like swimmers, and others have been described, suggesting a great versatility in applications [65].

The use of the magnetic field for the propulsion of nano-/microrobots is highly advantageous for several reasons: (a) nano-/microrobots can be wirelessly navigated; (b) no fuel is required; (c) collective behavior can be easily configured into swarming abilities, stimuli-related memory, origami-like architecture, etc.; (d) facile recollection and recyclability; (e) compatibility with other propulsion mechanisms that allow multiple modes of propulsion; and (f) good permeability through the environment [65]. On the other hand, the choice of magnetic materials is relatively poor, especially compared to light-activated nano-/microrobots [58], and magnetic navigation requires elaborate and costly setups [66].

##### Alternative field-driven nano-/microrobots

Alternative actuation mechanisms for nano-/microrobots have been developed: Nano-/microrobots can be propelled in acoustic [67,68] or electric fields [69]. These propulsion modes are specific, depending on the application and environment. The detailed mechanisms of the latter actuation mechanisms are not described in detail within the scope of this review.

#### Collective behavior and swarming abilities

In certain cases, a single nano-/microrobot cannot accomplish the desired task on its own. From this point of view, the researchers found inspiration in nature, where living organisms, such as bees or ants, gather and act as one body within swarms [70]. Nano-/microrobots with collective and swarming abilities can be used for various applications, particularly when the size of the pollutants exceeds the size of nano-/microrobots or when the capture and transport of pollutants is required in confined spaces [45]. The method of programming nano-/microrobots with collective behavior differs depending on the mechanism of swarm generation that can be triggered by light irradiation [71] and magnetic [72,73], electric [74], or acoustic fields [75].

Light-propelled nano-/microrobots can exhibit collective behavior owing to the generation of chemical and electrochemical gradients that induce subsequent diffusion and osmotic phenomena [76]. The generated phoretic forces cause the nano-/microrobots to cluster and expand in response to light irradiation, leading to the formation of pump-like systems. The generated phoretic forces can affect inert particles/pollutants, causing their capture by dynamic clusters of nano-/microrobots [77]. Alternatively, depending on the geometry, morphology, and material composition of nano-/microrobots, positive or negative phototaxis can be achieved to enhance the transport of pollutants or defined cargo [78,79]. A specific case of light-induced collective behavior is caused by thermophoresis induced by NIR light irradiation [80,81].

Collective behavior can also be induced in magnetic nano-/microrobots. Depending on the morphology and applied magnetic field, the nano-/microrobots can move collectively in synchronization in a specific manner (rolling, swinging) and direction [82]. In addition, the mode of the applied magnetic field can induce swarming behavior and/or magnetotaxis, which contributes significantly to the capture and transport of various substances and pollutants [83]. From this point of view, electromagnetic actuation systems, such as Helmholtz, Maxwell, or saddle coils, are necessary for navigation [84]. Moreover, nano-/microrobots can orient themselves on the basis of dipole–dipole interactions, forming various chains and ladder-like structures [85].

### Versatility of nano-/microrobots

Apart from propulsion abilities, nano-/microrobots must be programmed with an additional functionality to enable a desired task accomplishment. Functionality does not necessarily need to be only one; sophisticated systems are often required to be engineered (Fig. 2). The complexity of the structures is achieved because of the great versatility of materials, designs, morphologies, surface modifications, and their efficient combination. Depending on requirements, nano-/microrobots can be designed to capture, transport, release, degrade, convert, and catalyze, among others [30]. In addition, nano-/microrobots can communicate with each other, the environment, and the operator [86]. This is a large class of nano-/microrobots designed for sensing applications [87]. A certain level of intelligence can also be provided to nano-/microrobots by programming their actions and memory based on simple YES/NO operations [88]. With this approach, they can change their optical properties depending on changes in pH, or they can release cargo under specific temperature conditions [89]. Specific examples of environmentally directed applications of nano-/microrobots are discussed further.

In summary, the functionality and versatility of nano-/microrobots are achieved by the choice of materials and the chemical and physicochemical properties of the materials. However, the resulting architecture, such as morphology, symmetry, nature of binding, and interactions between the materials, must be considered as well because they also have a significant effect on functionality, propulsion abilities, and compatibility with the environment. From this point of view, the application of the concepts of nanoarchitectonics represents a powerful tool for the fabrication and functionalization of nano-/microrobots to assess the resulting functionality, abilities, and limitations.

## Environmental Remediation

As previously discussed, the application range of nano-/microrobots is broad—starting from medical application, bioapplications, environmental remediation, and not finishing at food industry or chemical conversions. Focusing only on environmental remediation processes, nano-/microrobots hold great potential as highly functional systems for various procedures, not only limited to detection, capture, transport, and degradation of pollutants, as discussed further [31].

### Remediation from different environments

Environmental remediation can be understood in different environments, meaning in different media, such as water, soil, and living organisms [16]. Each environment has certain specifics and limitations that must be reflected in the design of nano-/microrobots. At this point, it is relevant to repeatedly point out that a universal nano-/microrobot does not exist, and it always needs to be tailored for application purposes. One of the first criteria that should be considered before designing nano-/microrobots is the medium in which the specific task needs to be accomplished, as it will have an impact on the choice of the propulsion mode [90,91]. Focusing mainly on operation in aqueous environments, the propulsion of nano-/microrobots depends on the viscosity, pH, and ionic strength of the medium, in addition to the intrinsic properties of nano-/microrobots, such as size, morphology, and surface charge [48]. The most perceptive propulsion modes are those induced via ionic and electrochemical gradients that can be suppressed in the presence of different chemical substances and ions. Despite these limitations, there have been several reports introducing nano-/microrobots that operate in challenging environments, such as river water and sea water, and eventually in biological fluids such as urea, plasma, and blood [67,71]. Alternatively, challenging environments can also be represented by incoherent characters, such as gels, soil, or biological matrices that hinder motion through interfacial adsorption and friction [92]. In this regard, nano-/microrobots that work in the soil were designed to support the versatility of the concept of nano-/microrobotics in environmental remediation [93]. It should be noted that the complexity of the environment must be considered before designing propulsion mechanisms. In certain specific cases, the characteristics of the environment can provide efficient conditions for achieving propulsion abilities. For example, the use of H_2_O_2_ as a fuel during biofilm eradication is advantageous; as it provides disinfection properties by itself, it has a synergistic effect with nano-/microrobots on biofilm degradation [94].

### Mechanisms of remediation

**Fig. 4.**
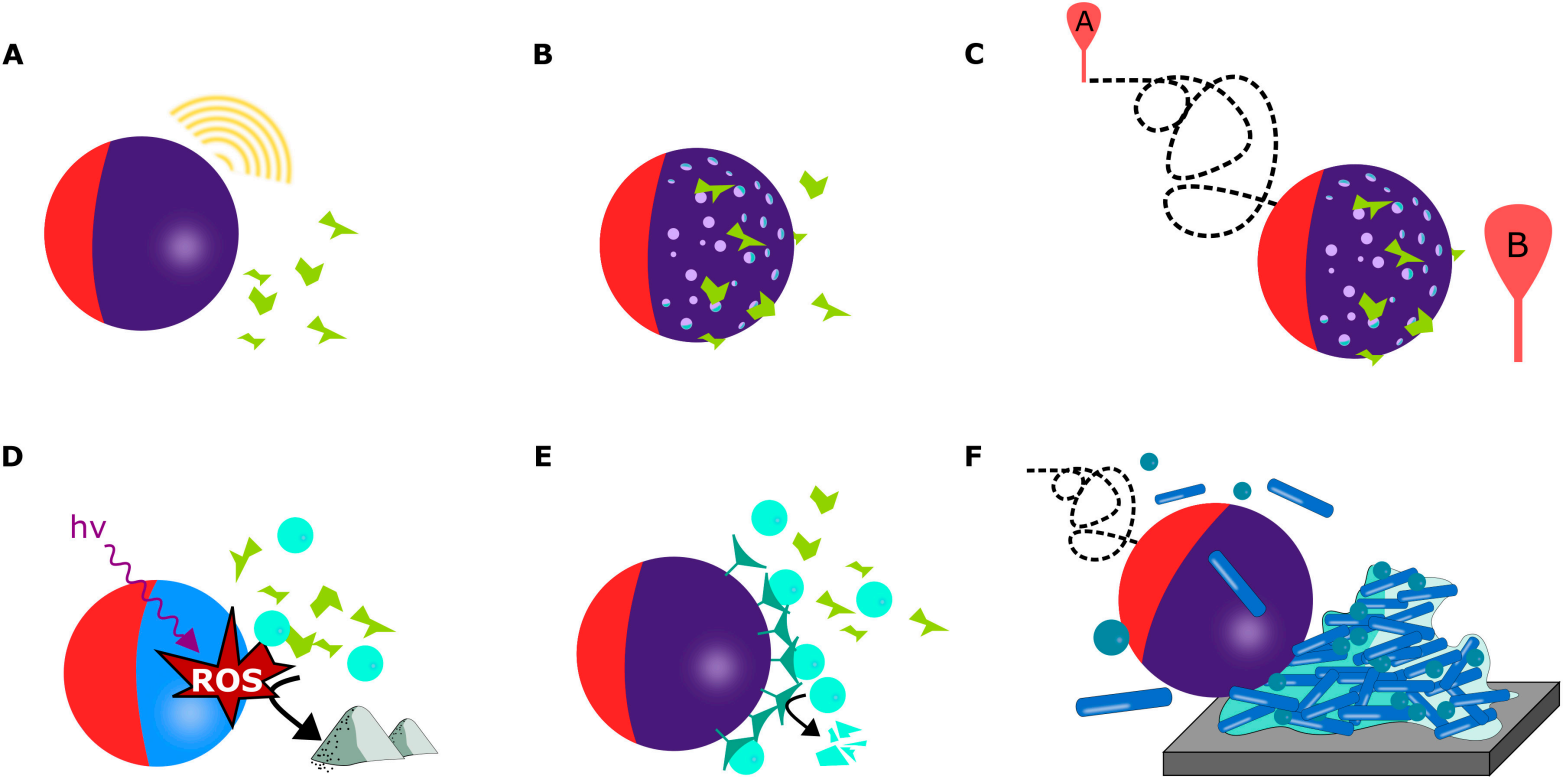
Schematic representation of nano-/microrobots in selected remediation processes. (A) Detection of pollutants. (B) Capture of pollutants over the porous structure of nano-/microrobots. (C) Transport of pollutants from the point of origin A to the point of collection or delivery B. (D) Nonselective photocatalytic degradation of pollutants by photogenerated reactive oxygen species (ROS). (E) Selective enzymatic degradation of pollutants. (F) Mechanical disruption of the biofilm.

Remediation can be understood as detection, quantification, capture, transport, in situ degradation of various pollutants, or a combination of these actions (Fig. 4). When considering environmental remediation, there are many different approaches to the elimination of the pollutants, such as toxic elements and ions, organic substances, particles, and/or microorganisms. Depending on the request, the design of nano-/microrobots must be tailored by designing the morphology, selection of material, and surface modifications, such as surface charge modification and decoration with nanoparticles, functional molecules, or single atoms [32].

#### Detection

The general concept of detection and sensing requires a specific and selective interaction with the analyte followed by signal generation. The main difference between static systems and nano-/microrobots developed for detection applications is their propulsion capabilities [95]. The propulsion of nano-/microrobots enables active pollutant detection in an “on-the-fly” mode, which increases the overall efficiency. Furthermore, nano-/microrobots with magnetic properties can be easily collected and eventually reused, thus increasing sustainability in remediation processes [96].

Typically, optical detection of pollutants is highly advantageous. Photoluminescence represents a powerful tool for detection via photoluminescence quenching, charge transfer, or wavelength shift induced by analyte binding. An additional advantage is that the required setup of spectrofluorometers, spectrometers, or readers is robust, available, and relatively fast, enabling fast action in the environmental remediation procedures. Furthermore, depending on the nature of the fluorophore, even low concentrations of pollutants can be efficiently monitored, allowing the detection of biological molecules, microorganisms, nano-/microplastics, toxic substances, and heavy metals [97].

#### Capture

Considering the capture of pollutants, the affinity between the pollutant and nano-/microrobots must first be achieved. This can be done nonspecifically via adsorption by choosing traditional adsorbent materials, such as zeolites, electrostatically by tuning surface charges or by exploiting hydrophobic–hydrophilic interactions. Alternatively, external fields can assist with the capture capability by inducing the collective behavior of nano-/microrobots [98]. For example, light irradiation can induce schooling behavior that can enhance capture efficiency upon photogenerated charge formation over the photocatalytically active body of nano-/microrobots via phoretic interactions [99]. Similarly, magnetic field can induce collective behavior, which can improve the efficiency of pollutant capture and transport. However, the interactions mentioned above are rather nonspecific and can influence substances that are not desired to be captured or removed from the system [100].

Capture selectivity can be increased by fine-tuning the surface chemistry using specific host–guest interactions and selective physical interactions, such as hydrogen bonding or enzymes. These systems are highly selective and efficient due to the contribution of the “on-the-fly” operational mode provided by the propulsion capabilities of nano-/microrobots [101].

#### Transport

To consider the transport of pollutants, the nature of the interaction with nano-/microrobots must be defined first, as transport can be combined with on-demand capture and release capabilities [102]. In different modes, pollutants can be collected from the environment, accumulated in a certain locality, and released or even degraded on demand. This concept offers reusability, which is generally considered a sustainable and environmentally friendly approach. It is also worth noting that swarming abilities and collective behavior are quite advantageous because swarms of nano-/microrobots can efficiently manipulate objects and pollutants larger in size than individual nano-/microrobots [103]. Taking into account the transport abilities themselves, they can be enabled using external fields via taxis, that is, magnetotaxis or phototaxis, which allows for precise navigation [104,105].

#### Degradation

One of the possible applications of nano-/microrobots in environmental remediation is in situ degradation of pollutants in the “on-the-fly” mode. Degradation can be achieved either (photo)catalytically or enzymatically. Photocatalytic degradation is the main application of semiconductor-based nano-/microrobots, which generate reactive oxygen species (ROS) upon light irradiation. ROS not only contribute to propulsion abilities as they induce phoretic forces but also enable photodegradation of organic pollutants [106,107]. From this perspective, organic pollutants such as food dyes, pesticides, hormones, and drugs can be efficiently degraded in situ. A specific example of ROS generation in the field of nano-/microrobotics is via Fenton reactions observed in iron-based materials. Iron containing nano-/microrobots corrode in the presence of H_2_O_2_ and release Fe^2+^ or Fe^3+^ ions, which subsequently generate ^•^OH radicals (Eqs. 9 and 10) that initiate degradation of organic pollutants and formation of various ROS [108].

$${\mathrm{Fe}}^{3+}+H_{2}O_{2}\to {\mathrm{Fe}}^{2+}+H^{+}+O_{2}H$$ (9)

$${\mathrm{Fe}}^{2+}+H_{2}O_{2}\to {\mathrm{Fe}}^{3+}+{\mathrm{HO}}^{-}+{\;}^{•}\text{OH}$$ (10)

From this point of view, αFe_2_O_3_ (hematite)-based nano-/microrobots appear to be highly efficient in pollutant degradation processes because they exhibit both photocatalytic properties and the ability to simultaneously provide Fenton reactions. Apart from that, both phenomena contribute to the propulsion capability [30].

It should be pointed out that photocatalytic degradation is nonspecific. The specificity to the desired pollutant degradation can be targeted by employing enzymes that can degrade pollutants in a certain manner by cleaving a specific chemical bond [109]. However, enzymes require precise control of the environment, including ionic strength, temperature, and pH. Additionally, they are prone to degradation, have a limited lifetime, and are costly.

#### Mechanical disruption

As nano-/microrobots operate in “on-the-fly” mode, their motion can be used to mechanically disrupt desired objects [110]. In particular, their application for the eradication of bacterial biofilms is highly advantageous [111]. Eradication is even more efficient when the photocatalytic nano-/microrobots are propelled in a light-induced manner that enables ROS generation. The ROS have been demonstrated to provide an antibacterial effect [112]. In addition, nano-/microrobots can be loaded with antibiotics or modified with silver to increase the bactericidal effect [42].

### Selectivity toward pollutants

The functionalities of nano-/microrobots show great applicability in environmental remediation. They have been applied for heavy metal remediation, toxic organic molecule detection, capture, and degradation, polymer and nano-/microplastic remediation, oil contamination treatment, microorganism remediation, and biofilm eradication. Although each application has quite the same task, the specific approach toward the engineering of nano-/microrobots can be different depending on the remediation mechanism, size and nature of pollution, character of the contaminated environment (i.e., presence of other substances, space confinement, state of matter, and others), accessibility of external sources for propulsion, and sensitivity toward chemical fuels that need to be applied for propelling nano-/microrobots. Given the multiple scenarios in the broad field of environmental remediation, the engineering of nano-/microrobots can be challenging. This is the point where nanoarchitectonics can take place and give a set of general rules and approaches toward precise engineering of the nanostructures that lead to efficient and selective task accomplishment.

## Nanoarchitectonics of Nano-/Microrobots for Environmental Remediation

This section reviews and summarizes the common approaches of nanoarchitectonics toward engineering of nano-/microrobots for environmental applications. To organize the general approaches in a logical way, the nanoarchitectonics at the nanoscale will be described first and subsequently extended to the level of quantum dots, individual molecules, and single atoms. This top-down approach is typically used in engineering multifunctional nano-/microrobots, where the main body represents the largest scale and provides the main functionality such as propulsion ability, affinity toward specific pollutants, semiconductive properties, or photocatalytic capability. Subsequently, additional fine and delicate modifications on the level of nanoparticles, quantum dots, and molecular and atomic level follow to design elaborate and complex functional systems.

### Nanoarchitectonics on the nanoscale

Nanoarchitectonics on the nanoscale is typically the first step in engineering functional nano-/microrobots. First, the size, morphology, and main material must be determined according to the desired task and the medium of operation. These parameters also have a crucial effect on the resulting propulsion abilities, monitoring possibilities, and versatility. Further nanoscale steps, such as surface coating to form Janus-type particles or decoration with functional nanoparticles or quantum dots, are required at the next stage.

#### Morphology control

The first crucial step in the engineering of nano-/microrobots is to determine the desired applications and specific tasks. This is followed by setting the dimensions and morphology along with programming the propulsion abilities of the nano-/microrobots.

Size significantly influences propulsion abilities and the application of nano-/microrobots. Mathematical models arising from Einstein–Stokes theory determine that the diffusion coefficient *D* is inversely proportional to the diameter of the particle, suggesting that nanosized swimmers can move faster than their microsized analogs [113]. Nevertheless, it should be noted that the resulting size of the nano-/microrobots must be considered with respect to monitoring capabilities. Although microrobots are easy to follow using optical microscopes and their swarms are visible to the naked eye, sub-100-nm nanorobots can be tracked only indirectly by advanced techniques, such as nanoparticle tracking analysis, which utilizes the properties of light scattering and Brownian motion [114].

Next, the locomotion mechanism and the resulting trajectories differ depending on the morphology [115]. These effects can be observed very well when designing magnetically navigated nano-/microrobots. Depending on the morphology, nano-/microrobots can walk, roll, jump, swim, wriggle, etc. [116]. In combination with the customization of size, highly functional magnetic field-driven nano-/microrobots can overcome obstacles arising from the microtopographical character of the substrate [117].

Morphology control is also important in the design of nano-/microrobots driven by diffusiophoresis or electrophoresis. Chemical fuel- or light-propelled nano-/microrobots with highly symmetric structures cannot be successfully propelled because of the homogeneous generation of chemical and electrochemical gradients. Breaking symmetry typically affects the directionality of motion. Villa et al. [115] synthesized single-component BiVO_4_ microrobots driven under visible light irradiation with 2 different morphologies: single 4-point and twin 4-point stars. Consequently, different trajectories of these morphologies were observed. While the single 4-pointed microrobots exhibited planar motion, the twin 4-pointed stars were able to stand up on the *Z* axis following the direction of the illumination. The numerical simulations supported the assumption that the symmetry of the microrobots played a crucial role when light was brought from one direction, causing the generation of an electrochemical gradient only from the illuminated side. From this point of view, it is desirable to break the symmetry in the case of chemically and light-driven nano-/microrobots. This can be achieved either by morphological tuning or by nonhomogeneous surface modification, such as the formation of Janus particles (as discussed below in more detail).

Finally, the size and shape of the nano-/microrobots have a significant influence on task accomplishment. Considering the capture and transport of pollutants, the size of nano/microrobots significantly affects their capture and eventual transport capabilities [118]. The size of the pollutant must be smaller than the size of nano-/microrobots. Eventually, the swarming abilities need to be provided to the nano-/microrobots to enhance the efficiency of the capture and transport abilities. Similarly, considering the capture of molecular or atomic-level contamination, smaller particles or porous structures are highly advantageous due to the increasing active surface availability [119].

#### Metallic layer coating

Metallic layer coating is traditionally performed as a step to break the symmetry in the morphology of nano-/microrobots, resulting in efficient propulsion. The most general concept is the fabrication of Janus particles by partial deposition of a platinum layer that catalyzes the decomposition of H_2_O_2_. In a different approach, the metallic layer can break the symmetry of photocatalytic nano-/microrobots and support charge separation under light irradiation. Eventually, the metallic layer can provide nano-/microrobots with additional specific properties, such as magnetic or antimicrobial properties [120,121].

##### Metal sputtering

Janus-like architecture is the most common type of nano-/microrobots’ design. In the most traditional approach, a layer of platinum is partially deposited over the main body of the nano-/microrobot to enable the catalytic decomposition of the H_2_O_2_ fuel. The partial coating brings nonsymmetry to the morphology; therefore, the generated chemical gradient over the platinum coating acts as the driving force for propulsion. In addition, providing the coating only partially does not hinder the entire active surface of the nano-/microrobot and allows further modifications or interactions with the environment [121].

**Fig. 5.**
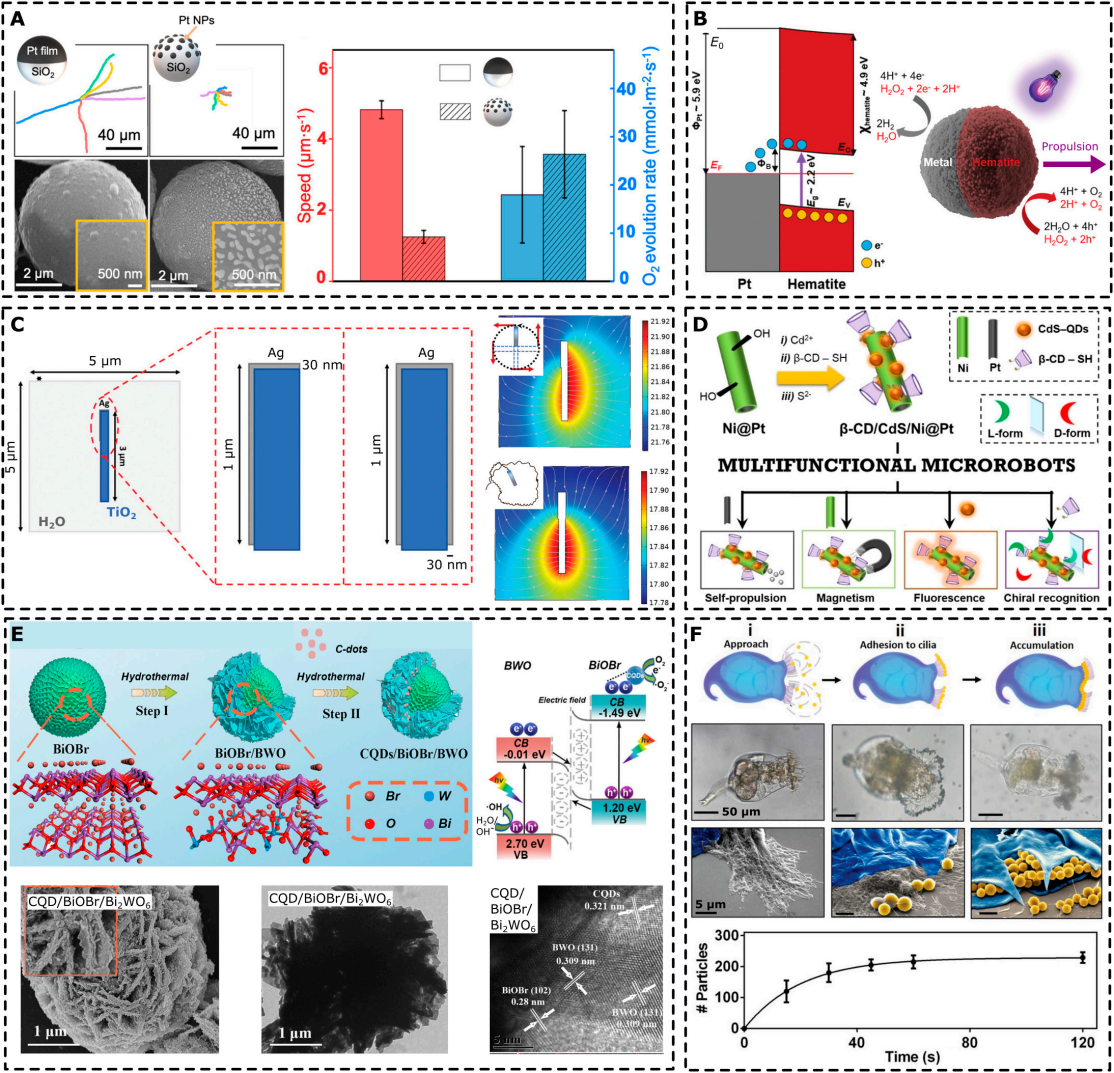
Nanoarchitectonics on the nanoscale. (A) Influence of the consistency of the platinum layer on the propulsion abilities of H_2_O_2_-driven microrobots. The plotted trajectories were observed for 20 s in the presence of 10 wt % H_2_O_2_. Adapted with permission from [50]. Copyright 2021, American Chemical Society. (B) Illustration of an electron transfer in hematite/Pt Janus microrobots under UV light irradiation; *E*_F_ is the Fermi level; *E*_0_ is the vacuum level; *E*_C_ and *E*_V_ are hematite conduction and valence band levels, respectively; *E*_g_ is the hematite optical bandgap; Φ_Pt_ is the Pt work function; Φ_B_ is the Schottky barrier. Adapted with permission from [127]. Copyright 2021, Wiley-VCH. (C) Influence of the symmetry of the silver coating on the propulsion abilities of black TiO_2_ nanorobots and numerical simulation of the spatial distribution of the H^+^ gradient of the corresponding configurations. The nanorobots were propelled under UV irradiation in the presence of H_2_O_2_ that was photocatalytically decomposed over a black TiO_2_ surface. Adapted with permission from [128]. Copyright 2022, Wiley-VCH. (D) Nanoarchitectonics of chiral-magneto-fluorescent microrobots designed for the enantiorecognition of chiral amino acids in the “on-the-fly” mode; β-CD refers to cyclodextrins. Adapted under terms of the CC BY license from [132]. Copyright 2022, Wiley-VCH. (E) Illustration of the preparation of BiOBr/Bi_2_WO_6_ (BWO) photocatalytic microrobots decorated with carbon quantum dots (CQDs) and corresponding electron microscopy images. The suggested charge transfer mode in the Z-scheme heterojunction is presented in the right upper corner; CB and VB refer to conduction and valence bands, respectively. Adapted with permission from [142]. Copyright 2022, American Chemical Society. (F) Nanoarchitectonics for the fabrication of Rotibods, including microscopy (scale bar, 50 μm) and scanning electron microscopy images (scale bar, 5 μm) at different stages, that is, (i) directional flow that attracts microbeads (yellow) to the rotifer’s mouth, (ii) adhesion of microbeads to the cilia, and (iii) accumulation of the microbeads in the cilia. The time dependence of the microbead accumulation is presented in the bottom graph. Adapted with permission from [145]. Copyright, 2019 Wiley-VCH.

It is worth discussing that the asymmetric decomposition of H_2_O_2_ over the body of Janus-type nano-/microrobots is not the only key parameter for a successful propulsion. Lyu et al. [50] conducted a complex study comparing different morphologies of platinum coatings on spherical SiO_2_/Pt Janus microrobots. The continuous coating of platinum on the caps of the spheres led to sufficient propulsion in the presence of H_2_O_2_ through a self-electrophoresis mechanism. When the cap consisted of a large array of platinum nanoparticles, the propulsion ability decreased dramatically, although the decomposition of H_2_O_2_ was more efficient than that of the film-coated Janus particles (Fig. 5A). The explanation comes from the theory that the propulsion mechanism is driven by electrophoresis (Fig. 3A). Assuming local thickness nonhomogeneities in the continuous platinum coating, thinner areas can act as anodes decomposing H_2_O_2_ into oxygen and 2h^+^ and 2e^−^, and due to the conductive character of the platinum cap, the charge is transferred to thicker cathode-like areas that generate H_2_O (Eqs. 3 and 4). Similar phenomena can also be assumed in the case of nanoparticles representing the cap of Janus microrobots. However, the electric and flow fields created over platinum nanoparticles cancel each other locally, eliminating the resulting propulsion ability. These results are in agreement with the propulsion driven by a self-electrophoretic mechanism rather than O_2_ bubble-induced propulsion in the case of H_2_O_2_-fueled nano-/microrobots.

A specific case of Janus-type nano-/microrobot fabrication involves modification of photocatalytic nano-/microrobots. Photocatalytic nano-/microrobots do not necessarily require modification with a platinum layer. They can be propelled under light irradiation because of the generation of electrons and holes that interact with water or fuel molecules and induce diffusiophoresis or electrophoresis (Fig. 3B) [122]. In this case, the main aim of Janus-type nano-/microrobot fabrication is to break the potential symmetry in the morphology and improve charge separation upon light absorption. When applying this approach in the engineering of nano-/microrobots, the synergy of H_2_O_2_ decomposition over platinum and over the semiconductor moiety can be achieved. Taking advantage of the different mechanisms behind fuel decomposition, propulsion abilities can be very precisely programmed. In this regard, Oral et al. [123] reported the propulsion modes of TiO_2_/Pt Janus microrobots with smooth and rough surfaces. Smooth TiO_2_ particles were coated with a continuous layer of platinum, while rough TiO_2_ particles did not allow continuous formation of a platinum layer. As a result, rough microrobots with a noncontinuous platinum coating exhibited propulsion in the presence of H_2_O_2_ owing to the catalytic decomposition of fuel over the platinum-decorated surface. However, when UV light irradiation was applied, the photocatalytic effects started to affect fuel decomposition throughout the whole body of the microrobots, and propulsion was no longer observed. On the contrary, smooth microrobots with discrete platinum coatings showed propulsion under dark conditions in the presence of H_2_O_2_, which was enhanced under UV light irradiation due to photocatalytic decomposition over the uncoated TiO_2_ body of the microrobot.

Similar observations have been reported for ZnO/Pt Janus microrobots [124]. In conclusion, a continuous metallic layer must be formed over the body of photocatalytic nano-/microrobots to achieve efficient charge separation for UV irradiation-induced propulsion. From this point of view, a smooth semiconductor–metal interface is required. This can be achieved by tailoring the morphology during the fabrication process of the main body of the nano-/microrobots, or by implementing additional interlayers that support charge separation. The latter was demonstrated in the work of Pourrahimi et al. [125], who sandwiched the interfacial layer of amorphous TiO_2_ between ZnO and platinum coating. The atomically thin amorphous TiO_2_ layer suppressed the Schottky barrier and enhanced charge separation, which allowed fuel-free propulsion under UV light irradiation.

One could argue that the surface roughness can be overcome by depositing a thicker layer of platinum. However, the thickness of the metallic layer can significantly influence the overall activity of the material of the main body of the nano-/microrobots. Peng et al. [126] reported a systematic study in which hematite spheres were decorated with platinum layers of 30, 60, and 90 nm to form Janus-type microrobots. As expected, micromotors with a thicker platinum layer exhibited higher speed due to the sufficient availability of metal for catalytic H_2_O_2_ decomposition. However, the increased thickness of the platinum layer hindered the photo-Fenton reaction on the hematite side of the microrobot.

Different metal coatings can be considered to adjust the electronic structure of photocatalytic nano-/microrobots to enhance charge separation under light irradiation. Urso et al. [127] conducted a comparative study in which hematite-based Janus-type microrobots were decorated with different metals, i.e., Au, Pt, Au–Pd, and Pt–Pd. Consequently, different metals influenced the electronic properties of the hematite composites and had a significant influence on the resulting speed of the microrobots in the presence of H_2_O_2_ fuel and UV irradiation (Fig. 5B). Moreover, the overall photocatalytic abilities were significantly affected, as demonstrated by the photocatalytic degradation of the polymer chains.

Metal coating can also result in broadened functionality, in addition to bringing asymmetry and improving charge separation in the design of nano-/microrobots. Ussia et al. [128] modified TiO_2_ nanorobots with silver coating. The silver coating broke the symmetry in the generation of ionic species, resulting in an efficient propulsion mechanism. Depending on the coating geometry, the resulting trajectories of the nanorobots differed. When the silver layer was sputtered on the tips of the nanotubes, the nanorobots exhibited random motion. However, when the silver layer was deposited mostly on one side of the nanotube, the nanorobots exhibited rotational trajectories (Fig. 5C). Additionally, the silver surface partially corroded and released Ag^+^ ions in the presence of H_2_O_2_ fuel and under UV light irradiation, which provided the nanorobots with bactericidal properties during the eradication of biofilms.

Alternatively, Janus particles can be formed by subsequent layers of nickel and gold (Fig. 5D). In this case, the nickel coating brings the required asymmetry and simultaneously the ability to navigate in a magnetic field, and the gold layer prevents the oxidation of the nickel layer [129,130].

##### Templating

Decoration with metallic layers can also be performed electrochemically via templating. To fabricate tubular nano-/microrobots, often referred to as microrockets, porous membranes can be used as templates that allow subsequent deposition of different layers to fabricate multifunctional nano-/microrobots. In a common arrangement, the outer layer of tubular nano-/microrobots is deposited first and it provides the main surface functionality of the body of nano-/microtubes. It can provide the resulting nano-/microrobots with photoresponsive abilities or a functionalized surface available for additional interactions. Subsequently, a layer of nickel can be deposited to provide the resulting nano-/microrobots with magnetic properties. Finally, the inner layer can be formed with platinum to allow chemical fuel-driven propulsion for the “on-the-fly” action (Fig. 5D) [131,132].

#### Self-aggregation and self-assembly

In a different setting, nano-/microrobots do not need to be decorated with metallic layers to bring asymmetry to the structure. In fact, the use of costly metals (such as platinum, gold, and silver) and the use of specific equipment, such as sputtering devices, are not economical from a mass-scale point of view. Semiconductor-based materials, such as TiO_2_ and ZnO, among others can provide the resulting nano-/microrobots with propulsion abilities when designed in a suitable morphology. In the eyes of nanoarchitectonics, the use of self-aggregation and self-assembly abilities followed by Ostwald ripening to form a hierarchical structure is an easily scalable and economical solution for nano-/microrobot fabrication [133,134]. Moreover, the self-assembly process can be monitored using capping agents that form complexes with precursor salts that can couple via nonchemical bonding to organize and control the material growth and resulting morphology. These processes are typically performed by using hydrothermal methods. A recent study demonstrated great efficiency and scalability using a microwave reactor for the hydrothermal preparation of nano-/microrobots with reduced reaction times [135].

#### Nanoparticle and quantum dot decoration

For nanoscale nanoarchitectonics, decoration with nanoparticles and quantum dots must be mentioned. The main advantage of using nanoparticles and quantum dots for the decoration of nano-/microrobots is the possibility of decorating the surface with additional functionality while keeping the material of the main body available for other interactions or functionalization.

##### Nanoparticles

Nanoparticle attachment is typically achieved via electrostatic interactions. A typical example is the use of magnetic nanoparticles for the decoration of nano-/microrobots to achieve magnetic field-driven navigation. Consistent with this concept, Wang et al. [136] designed a polycaprolactone (PCL)-based microrobot decorated with iron oxide nanoparticles. Surface availability enabled additional modification with catalase to enable propulsion in the presence of H_2_O_2_ as fuel to achieve dual mode propulsion. In addition, the availability of the PCL surface resulted in the fabrication of hydrophobic microrobots that enabled affinity toward oil capture and removal. Similarly, Beladi-Mousavi et al. [137] decorated BiVO_4_ microrobots with magnetic nanoparticles that enabled magnetic manipulation of the microrobots while maintaining the photocatalytically active surface available for the adsorption and degradation of various microplastics. It should be highlighted that a common feature demonstrated in the 2 latter studies is that the nano-/microrobots are propelled in dual modes. Fuel-driven propulsion enables the achievement of autonomous task in the “on-the-fly” mode, while magnetic navigation allows the subsequent recovery from the environment and recycling procedures.

Nanoarchitectonics allows the use of different nanoparticles that can help to achieve precisely designed multifunctional nano-/microrobots. For example, Gong et al. [138] decorated Spirulina cells with MnO_2_ nanoparticles along with magnetic nanoparticles. The magnetic nanoparticles provided the ability to navigate the microrobots in a magnetic field, and the MnO_2_ nanoparticles provided adsorption activity toward heavy metal pollution. Alternatively, Ussia et al. [139] loaded ZnO microrobots with silver nanoparticles during a one-step hydrothermal preparation to achieve higher charge separation in ZnO to propel microrobots in H_2_O_2_ under UV light irradiation through a self-electrophoresis mechanism. In addition to the influence on propulsion abilities, the presence of silver nanoparticles provided the resulting microrobots with antibacterial activity that enhanced biofilm eradication.

##### Quantum dots

The application of quantum dots is often associated with their unique tunable optical and electronic properties. In the field of nano-/microrobotics, quantum dots can especially contribute with their optical properties, such as photoluminescence and its related quenching processes in sensing applications [140]. For example, PbS quantum dots have been applied to dope Cu_2_O microrobots in a one-pot reaction to achieve NIR fluorescence [141].

The presence of quantum dots can tailor the electronic properties of nano-/microrobots. Zhang et al. [142] designed BiOBr/Bi_2_WO_6_ microrobots decorated with carbon quantum dots. The precise architecture led to the generation of a Z-scheme heterojunction, where carbon quantum dots enhanced the charge transfer between BiOBr and Bi_2_WO_6_ (Fig. 5E). The resulting microrobots were propelled under visible light via a self-diffusion electrophoresis mechanism, and their photocatalytic abilities were evaluated by demonstrating the photocatalytic degradation of the model antibiotics.

In addition to fluorescence and electronic properties, quantum dots can also bring advanced functionality to nano-/microrobot systems. One such example could be found in the study of Jurado-Sánchez et al. [140] who modified poly(3,4-ethylenedioxythiophene) (PEDOT)/poly(sodium 4-styrene sulfonate) (PSS)-based microrockets with CdTe quantum dots. As direct attachment of CdTe quantum dots was not possible on the PEDOT/PSS surface, a positively charged poly(diallyldimethylammonium chloride) (PDDA) layer was deposited via the LBL method to provide a positive surface charge that electrostatically interacted with the –COOH functional groups of CdTe quantum dots. The resulting microrobots exhibited the ability to selectively bind Hg(II) species owing to their interaction with CdTe, which was visualized via photoluminescence quenching processes. This concept enabled the successful in situ detection and capture of heavy metals.

#### Biotemplating

A special approach in the field of nanoarchitectonics toward the engineering of nano-/microrobots is biotemplating. Various natural biotemplates, such as microorganisms, can be used as templates during the fabrication process, or they can be directly modified into microrobots. The biotemplating approach seems to be advantageous because the use of bio-originated materials limits the use of artificial and potentially hazardous materials. Furthermore, the versatility of microorganisms can be exploited in the field of nanomicrorobotics [143]. With regard to this concept, helical microrobots were derived from spirulina by decorating algae cells with magnetite nanoparticles and subsequent vacuum annealing to achieve a hollow core-shell structure [144]. In this design, microrobots were used to remove Cr(VI) via an adsorption mechanism. Moreover, cytotoxicity toward *Escherichia coli* was observed owing to the photothermal attributes of magnetite nanoparticles and amorphous carbon. Similarly, Peng et al. [118] derived microrobots from algae *Chlorella vulgaris* and decorated them with magnetic nanoparticles through electrostatic interactions between the nanoparticles and the negatively charged algal cytomembrane. The nature of the algae provided the resulting microrobots with a high electrostatic affinity toward the model polystyrene nano-/microplastics.

In an alternative approach, microorganisms do not need to serve as inactive templates. They can be modified to perform a specific task while remaining alive. Soto et al. [145] designed the so-called Rotibods—biohybrid microrobots derived from marine rotifers (*Brachionous*) (Fig. 5F). The cilia of Rotibods were electrostatically functionalized with lysozome and organophosphorus hydrolase enzymes. The microorganism itself acted as a motor and induced active flow of the contaminated liquid around the lysozome and enzyme-decorated areas, leading to the degradation of *E. coli* and the nerve agent methyl paraoxon. In addition, remediation of heavy metal contamination was possible.

To conclude the overall findings from the “Nanoarchitectonics on the nanoscale” section, the nanoarchitectonics on the nanoscale has a direct impact on propulsion abilities, main functionality, and functionalization possibilities of nano-/microrobots in environmental applications. Following the top-down trend, the size and morphology have a direct influence on the propulsion abilities in desired environments. Simultaneously, the size and morphology (including symmetry and overall shape, surface area, and additional decorations with nanoparticles and quantum dots) of nano-/microrobots significantly affect the interaction with pollutants, i.e., the size of nano-/microrobots must be considered when aiming for capture and transport of microscopic pollutants, the surface area must be maximized in case of heavy metal adsorption, and/or availability of photocatalytic surface must be ensured for efficient photocatalytic activity. Apart from size and morphology, electronic properties of the materials used for the fabrication of nano-/microrobots can be tuned by modifications on nanoscale by metal coating and decoration with nanoparticles or quantum dots. This approach is crucial especially for photocatalytically active nano-/microrobots that are typically employed for light-driven propulsion and photocatalytic degradation of targeted pollutants.

### Nanoarchitectonics at the interface

**Fig. 6.**
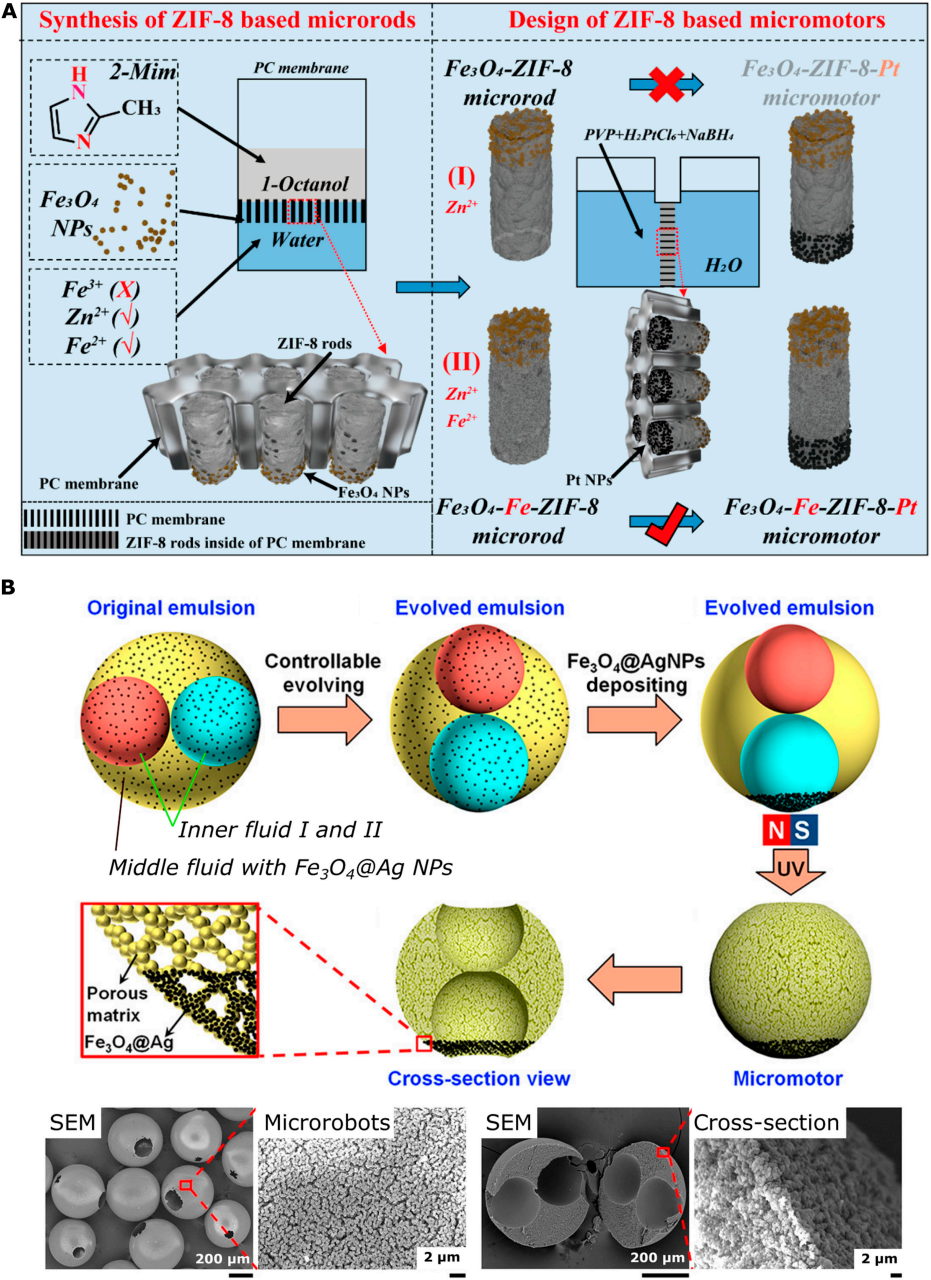
Nanoarchitectonics at the interface. (A) Illustration of the fabrication of magnetic ZIF-8 microrobots at the interface using a porous polycarbonate (PC) membrane as a template. Adapted with permission from [146]. Copyright 2019, American Chemical Society. (B) Nanoarchitectonics of porous micromotors fabricated from evolved double emulsions and scanning electron micrographs of the resulting microrobots. Adapted with permission from [148]. Copyright 2019, American Chemical Society.

Nanoarchitectonics at the interface is represented by a specific set of tools that operate laterally at the level of single molecules with lateral extensions to the nanoscale [27]. The interface can be of different origin. It can be liquid–liquid, liquid–air, and solid–liquid, resulting in environments of various specifics and features. Nevertheless, a common feature is the specific and organized assembly of individual molecules at the interface into functional and organized entities. A high degree of organization can be brought to a limited area by templating, as demonstrated by Ying et al. [146], who fabricated ZIF-8-based microrobots at the interface (Fig. 6A). To limit the lateral dimensions, fabrication was performed using a polycarbonate membrane as a template. Subsequently, the ZIF-8 material was decorated with magnetic particles and partial platinum coating to enable propulsion in the presence of H_2_O_2_. The precise architecture enabled the removal of uranium from the environment in the “on-the-fly” mode.

Confinement of the lateral dimensions can also be achieved by forming emulsions. Qiu et al. [147] fabricated mesoporous silica nanorobots using a multistep approach. First, silica shells were fabricated from a water/*n*-pentanol emulsion system that organized silica precursors at the interface into bottleneck-like nanorobots. The nanorobots were subsequently decorated with Fe_3_O_4_ nanoparticles by in situ growth on their surface. In the last step, an ordered mesoporous silica layer was deposited using a biphase stratification assembly approach. Applying the principles of nanoarchitectonics, the hydroxyl-rich silicon surface enabled the attachment of catalase via hydrogen bonding, enabling self-diffusiophoresis-driven propulsion in the presence of H_2_O_2_. Because of the mesoporous character, the nanorobots were tested for heavy metal capture. In this study, Cu^2+^, Pb^2+^, and Cd^2+^ ions were collected from model aqueous environments.

The architecture of nano-/microrobots can be precisely tuned by adjusting the nature of the emulsion. Su et al. [148] fabricated hierarchical porous micromotors from controllably evolved double emulsions. In this setting, the resulting spherical microrobot contained 2 aligned microscale pores (Fig. 6B). Furthermore, to achieve nonhomogeneity, the bottom opening surface was decorated with Fe_3_O_4_@Ag nanoparticles, which enabled H_2_O_2_-fueled motion. The accumulation of magnetic particles at the specific end was achieved by applying an external magnetic field that forced the nanoparticles to accumulate at a specific position during UV light-induced polymerization of ethoxylated trimethylolpropane triacrylate (EPTA) in the middle fluid. The overall porous structure enabled the efficient capture of oil and heavy metal pollutants. Similarly, Jurado-Sánchez et al. [149] fabricated microrobots using self-assembled PCL polymers, platinum nanoparticles, iron oxide nanoparticles, and graphene quantum dots within the chloroform phase in water emulsions. Owing to the insolubility of platinum and iron oxide nanoparticles in PCL, they accumulated nonhomogeneously on one side of the spheres during the evaporation of chloroform, thus forming Janus-type microrobots.

To conclude, nanoarchitectonics at the interface provides a set of tools for lateral operations to organize precise structures with a high hierarchy. Such structures are often required when fabricating nano-/microrobots of defined morphology. In those cases, the interfaces are used as templates to control the size, morphology, and structure of the resulting nano-/microrobots. These methods are traditionally involved when hollow or porous systems are needed to increase active surface availability for the capture of pollutants, such as heavy metal ions.

### Nanoarchitectonics on molecular level

Finer tuning of nano-/microrobots toward advanced functionality can be achieved by their modifications on molecular and atomic levels. Nanoarchitectonics at the molecular level can be achieved via nonbonding interactions, such as molecular self-assembly, hydrogen bonding, electrostatically, or chemically, by linking the body of nano-/microrobots with a functional molecule via a chemical bond. An overview of the different approaches to nanoarchitectonics at the molecular level is presented in this section.

#### Polymerization

The use of polymers in the fabrication of nano-/microrobots is advantageous. First, polymers possess a robust character and, depending on their chemical nature, multiple functionalities can be achieved, such as soft nano-/microrobotics, stimuli-responsive transformations, and surface functionalization [37,150]. As the chemistry and versatility of polymers can be immense, we present several examples to give the reader an idea of the possibilities and approaches toward the fabrication of advanced functional nano-/microrobots.

Polymers can act as active components for designing the propulsion abilities of nano-/microrobots. Kochergin et al. [151] polymerized a semiconducting conjugated polymer triazine–thiophene diacetylene-bridged network over amine-grafted silica microtubes with Cu^+^ ions through Glaser-type polycondensation. The photocatalytic properties of the polymer enabled propulsion of tubular micromotors under visible light in the absence of H_2_O_2_ via self-diffusiophoresis. Simultaneously, the generated ROS were available for “on-the-fly” photodegradation of the psychoactive amphetamine-type drug 3,4-methylenedioxymethamphetamine (MDMA).

As previously noted, electrodeposition is of special importance in the template-assisted fabrication of multilayered tubular nano-/microrobots. In this regard, electropolymerization is a convenient and precise method to modify the surfaces of nano-/microrobots. An example could be the electropolymerization of polypyrrole in the pores of a membrane, which acts as a matrix for the fabrication of tubular microrobots. Subsequent electrodeposition of a platinum layer enables propulsion in the presence of H_2_O_2_ fuel. Tubular microrobots can be subsequently decorated with magnetic nanoparticles to enable magnetic navigation. In this specific design, microrobots were described to have the ability to concentrate 17α-ethynylestradiol (EE2) hormone pollutants and induce their filament formation. The mechanism of the hormone net formation emerged from the protonation of the polypyrrole layer under acidic conditions, which concentrated the hormone in its direct proximity [152].

Hou et al. [153] reported a similar design for tubular microrobots templated in a porous membrane. The outer functional layer was fabricated by electropolymerization of asparagine and cysteine. Subsequently, nickel and platinum layers were deposited to enable magnetic navigation and propulsion in the presence of H_2_O_2_, respectively. The surface based on polyasparagine-cysteine copolymers exhibited great ability to capture inorganic and organic heavy metal contamination in aquatic solutions due to the availability of surface carboxyl, amino, and thiol groups.

#### Molecular self-assembly

Multifunctional molecules can be assembled within the main body of nano-/microrobots, or they can eventually form the main body of the nano-/microrobots. The advantage of forming molecularly self-assembled structures is the control of the resulting morphology. In addition, when organic molecules with specific functional groups are incorporated, additional decoration or enhanced specific pollutant remediation is enabled. In general, molecular self-assembly is driven by intermolecular interactions such as electrostatic interactions, hydrogen bonding, hydrophilic–hydrophobic interactions, and π–π stacking. Different molecular self-assembly mechanisms are discussed in the following.

##### Hydrogen bonding

A specific class of self-assembly ability is the hydrogen-bond-induced self-assembly of nano-/microrobots. Hydrogen bonding is a very specific type of interaction that can efficiently organize targeted molecules into highly ordered structures at the nano- or even microscale; however, it requires a very precise molecular architecture. Hydrogen bonding-induced self-assembly has been used for the fabrication of microrobots from organic hydrogen-bonded pigments [154]. The resulting microrobots exhibited highly hydrophobic properties that allowed the capture and collection of oil contamination.

##### LBL deposition

LBL deposition is a technique that allows the formation of molecularly thick layers that are organized on the basis of the interactions between them. Repeated coating over sacrificial particles allows for the formation of microrobots of precisely designed morphology, as reported by Wu et al. in 2012 [155]. Recently, the LBL deposition of a metal-phenolic network over polystyrene sphere templates to obtain hollow structures was presented by Guo et al. [156]. First, LBL deposition was performed to coat the entire sacrificial polystyrene sphere, followed by an additional partial surface coating to introduce nonhomogeneity into the morphology. The resulting microrobots were propelled photothermally under NIR light and were proposed as radical scavengers for antioxidative treatment.

With the concept of LBL deposition, various structures can be obtained by carefully selecting different templates. For example, the group of He [157,158] designed microrockets by LBL deposition of functional polymer within the sacrificial template membrane.

##### Intermolecular self-assembly

**Fig. 7.**
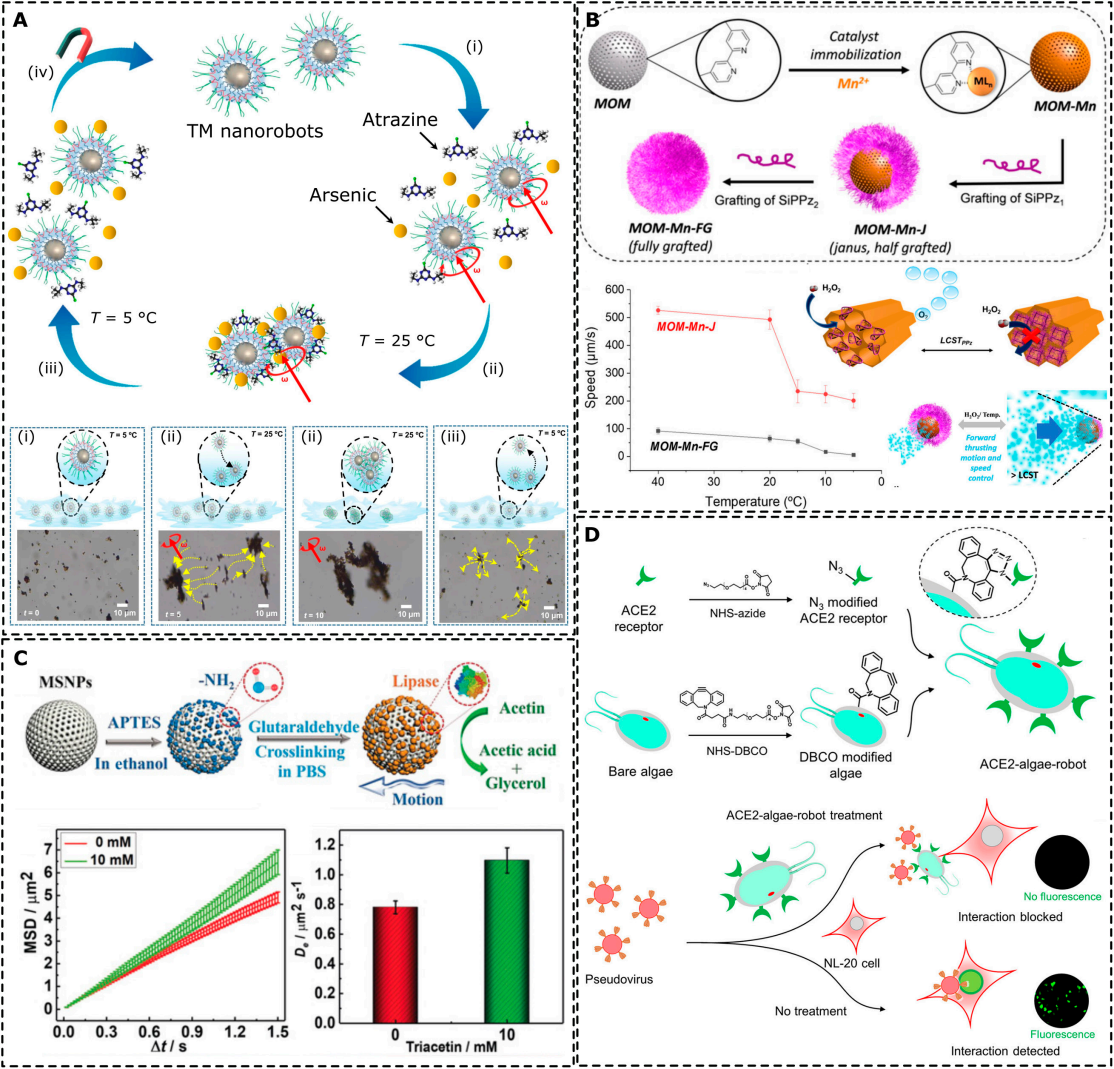
Nanoarchitectonics on the molecular level. (A) Operating mode of thermosensitive magnetic nanorobots (TM nanorobots) demonstrating attachment of pollutants (i), their subsequent capture at elevated temperature (ii), on-demand release at reduced temperature (iii), and magnetic collection toward reuse (iv). Adapted under terms of the CC BY license from [159]. Copyright 2022, Springer Nature. (B) Nanoarchitectonics of mesoporous organosilica micromotors (MOMs) and their decoration with polyphosphazenes SiPPz*_i_*, where *i* represents polymers of different molecular weight. The bottom part illustrates the dependence of speed on the temperature of the environment in the presence of H_2_O_2_. LCST refers to a lower critical solution temperature. Adapted with permission from [164]. Copyright 2021, Wiley-VCH. (C) Design of lipase-functionalized mesoporous silica nanorobots and demonstration of propulsion abilities in the presence of 10 mM triacetin, which acts as chemical fuel. Adapted with permission from [168]. Copyright 2019, Wiley-VCH. (D) Nanoarchitectonics of algae-based microrobots for removal of the SARS-CoV-2 virus from wastewater. The reaction scheme illustrates a click chemistry approach to conjugating the angiotensin-converting enzyme 2 (ACE2) receptor to the surface of the algae by the reaction between the azide and dibenzocyclooctyne (DBCO) groups. The mechanism of capture of the SARS-CoV-2 pseudovirus is represented in the bottom part. Adapted with permission from [169]. Copyright 2021, American Chemical Society.

Self-assembly mechanisms can occur dynamically over the surface of nanorobots. This approach was demonstrated by Vaghasiya et al. [159], who decorated magnetic microrobots with a pluronic triblock copolymer that shows a reversible temperature-induced phase transformation in an aqueous environment (Fig. 7A). Below the critical micelle temperature, the copolymer is dispersed in an aqueous environment in its free form; on the contrary, exceeding the critical micelle temperature induces precipitation. These dynamics can occur on the surface of nanorobots decorated with the immobilized copolymer. At temperatures below 25 °C, the copolymer chains expanded and, upon increasing the temperature, the polymer exhibited strong interactions, leading to shrinkage and capture of pollutants such as heavy metals or pesticides. Notably, the capture was reversible, allowing good recovery retention after multiple reuse cycles.

#### Surface decoration

The surface functionality of the nano-/microrobots can be attributed to the nature of the material that represents the main body, which is typically photodegradation abilities or pollutant adsorption capabilities. In some cases, further broadening of the functionality is required to achieve specific applicability of the nano-/microrobots. To tune the fine and delicate properties of nano-/microrobots, nanoarchitectonics with a set of surface chemistry approaches must be employed. The nature of the surface predetermines the methods of attachment of the desired advanced molecules, which can be of synthetic origin (smart polymers, molecular probes, etc.) [37,97] or bioinspired (enzymes, nucleic acids, etc.) [160]. In general, smart molecules can be attached noncovalently or covalently by forming chemical bonds. A special case of covalent bonding is the click chemistry approach, which provides a pool of different functional groups that can bind chemically with a high yield under moderate conditions. It is worth noting that the character of the binding should be considered carefully; chemical bonds provide great stability during the operation, whereas noncovalent binding can lead to washing out the surface molecules, thus decreasing the possibility of reuse. On the other hand, under controlled conditions, on-demand release can be achieved by noncovalent binding of the targeted molecules.

##### Noncovalent interactions

Depending on the nature of the noncovalent interactions, the attachment of functional molecules can be selective or nonselective. High selectivity is achieved by employing interactions such as hydrogen bonding or π–π stacking, which require specific configurations of the molecules to be bound. Furthermore, because hydrogen bonding and π–π stacking require precise architecture and organization of the structures, highly ordered systems can be formed and, in some cases, assist with charge separation or signal transfer. An example of π–π stacking-induced surface decoration was recently reported by Jyoti et al. [161]. The tubular microrobots were electrochemically decorated with graphene quantum dots, allowing the adsorption of a probe labeled with fluorescein amidite (FAM-L) via π–π stacking between the structure of the probe and the sp^2^-rich skeleton of graphene quantum dots. The character of π–π stacking not only allowed decoration itself but also enabled dynamics between the probe and quantum dots during photoluminescence quenching upon interactions with a complementary DNA sequence with the FAM-L probe.

A lower selectivity for functional molecule binding is achieved through electrostatic interactions and adsorption mechanisms. In general, these approaches are universal because they do not require any alignment of the functional groups or organized molecular architectures. Nevertheless, a certain degree of surface suitability must still be ensured, such as an appropriate surface charge to allow decoration. From this point of view, there are various materials that provide the ability to electrostatically bind various species. Polydopamine is a commonly chosen material owing to its adhesive properties. It can be applied to coat magnetic nanorobots via a self-polymerization procedure [162]. In this design, the resulting nanorobots were suitable for microplastics capture. Additionally, the polydopamine surface allowed electrostatic immobilization of the lipase enzyme, which enabled in situ microplastic enzymatic degradation.

In the case of an unsuitable surface charge that does not allow electrostatic interaction with a specific moiety, interlayers that change the surface charge can be deposited on the nano-/microrobots. Following this nanoarchitectonics methodology, magnetic microrobots derived from the mesoporous tea buds of *Camelia sinensis* were coated with cationic chitosan to modify the surface with a positive charge, which allowed the successful adsorption of antibiotic molecules. Subsequent desorption in an acidic environment allowed for on-demand drug release. The concept of microrobots has been suggested for the disruption of bacterial biofilms. In this setting, the microrobots provided a complex solution for biofilm eradication because 3 tasks were accomplished: (a) killing bacteria with antibiotics, (b) mechanically disturbing biofilm to enhance the access of drug, and (c) magnetically driven removal of the degraded biomass that was attached to the surface of microrobots [163].

##### Molecular recognition

A specific noncovalent interaction mentioned in this section is represented by molecular recognition. Molecular recognition allows for very selective binding of various analytes. Selectivity is traditionally achieved because of the molecular architecture and orientation via highly selective binding, such as hydrogen bonding, metal coordination, hydrophobic forces, van der Waals forces, and π–π interactions. Smart systems for detection, sensing, and selective remediation can be achieved by providing nano-/microrobots with moieties available for molecular recognition. In this regard, the work of Munoz et al. [132] should be mentioned where the decoration of tubular microrobots with β-cyclodextrin enabled chiral recognition capabilities toward tryptophan amino acid in the “on-the-fly” mode (Fig. 5D).

##### Covalent immobilization

Linking the functional molecule to the main body of the nano-/microrobot via a chemical bond has a significant impact on the stability of the system. Unless the bond is prone to specific degradation under certain conditions, no washout or surface dynamics occur. Functional molecules can be attached directly through available functional groups or by linkers and crosslinkers.

Covalent immobilization of thermoresponsive polyphosphazenes in mesoporous organosilica microrobots was reported to regulate propulsion abilities depending on temperature (Fig. 7B) [164]. Immobilized polyphosphazenes undergo conformational changes at different temperatures, which enables control of the accessibility of fuel to the catalytic surface of microrobots. The decoration of microrobots with polyphosphazenes was done in 2 configurations: first over the whole surface and then partially to fabricate Janus-type microrobots. As expected, the nonhomogeneity of the Janus-type microrobots resulted in efficient propulsion abilities controlled via temperature changes.

A commonly applied immobilization approach involves attaching thiol-containing molecules to gold surfaces. The Au–S interaction is ubiquitous across surface chemistry, since it proves to be highly versatile; it can also be applied in the field of nano-/microrobotics. Wang et al. [165] decorated Au/Pt microrobots with polyT30-SH oligonucleotides to fabricate DNA-decorated microrobots. Because of the presence of thymine moieties in the DNA oligonucleotide, an affinity for Hg(II) was achieved, providing a solution for the removal of heavy metal contamination from aqueous environments in the “on-the-fly” mode.

Alternatively, low molecular weight linkers, such as 1-ethyl-3-(3-dimethylaminopropyl)carbodiimide (EDC)/*N*-hydroxysuccinimide (NHS), can be used for chemical linking of functional molecules. EDC/NHS linkers are traditionally used for surface decorations with enzymes. Uygun et al. [166] fabricated tubular microrobots by electropolymerization of COOH-pyrrole and EDOT with the assistance of a porous membrane that acted as a template. The outer surface of the microrobots possessed COOH groups that were used to attach the laccase enzyme through EDC/NHS linkers to enable the enzymatic degradation of dye molecules.

The use of EDC/NHS linkers is not strictly limited to the attachment of biomolecules. For example, Ussia et al. [167] modified commercial magnetic nanoparticles decorated with amino groups by attaching a poly[*N*-(3-(dimethylamino)-propyl]methacrylamide polymer via EDC/NHS linkers to provide the resulting magnetic nanorobots with the ability to electrostatically capture pollutants, such as bacteria and microplastics.

An additional approach involves crosslinking the functional molecules on the active surface. In this scenario, glutaraldehyde is often used to crosslink the amino groups of the corresponding polypeptides. Wang et al. [168] decorated mesoporous silica nanoparticles with lipase using a glutaraldehyde crosslinker (Fig. 7C). The presence of lipase enabled chemically driven propulsion by the decomposition of triglycerides. The decomposition of lipids was aimed not only to achieve propulsion abilities but also to degrade blood lipids in organisms. Using this concept, efficient systems for the remediation of oil pollution can be designed.

##### Click chemistry

Click chemistry represents a specific approach toward the chemical immobilization of functional molecules on desired moieties. The general tools of click chemistry represent a straightforward way of chemically linking 2 moieties with high yields, fast reaction rates, and minimal byproducts. These approaches are also convenient for the nanoarchitectonics of nano-/microrobots. Zhang et al. [169] fabricated a biohybrid microrobot based on *Chlamydomonas reinhardtii* algae decorated with dibenzocyclooctyne-*N*-hydroxysuccinimidyl ester (NHS-DBCO) through a click chemistry approach to modify the angiotensin-converting enzyme 2 (ACE2) receptor that binds specifically to the severe acute respiratory syndrome coronavirus 2 (SARS-CoV-2) spike protein (S protein) (Fig. 7D). Modifications did not affect the self-propulsion abilities of algae, thus allowing successful “on-the-fly” capture of viruses from the aqueous environment.

In conclusion, nanoarchitectonics at the molecular level offers precise design of nano-/microrobots that originates from molecular interactions. The control of the size and morphology of nano-/microrobots can be achieved by employing the bonding or nonbonding molecular interactions, including polymerization, molecular organization, and self-assembly processes. As these processes are driven on the molecular level, the resulting structures are highly organized and provide the nano-/microrobots with precise structural hierarchy and defined optoelectronic properties that are crucial in designing propulsion, pollutant capture, and degradation abilities. Moreover, nanoarchitectonics at the molecular level enables surface modifications to tailor the nano-/microrobots for specific purposes; the molecular level decorations bring selectivity toward desired analytes and enable advanced sensing and elimination of viruses, selective degradation of oil pollutants, chiral recognition, and others.

### Nanoarchitectonics on the level of single atoms

The necessity to miniaturize various kinds of devices while maintaining functionality to the maximum possible extent is a general trend since the field of nanotechnology was postulated. Exploiting the functionality at the level of single atoms not only provides space for the miniaturization of the resulting functional systems but also significantly affects the economy of the resulting processes: The isolated atomic-level functional centers allow full access to the analytes and the material used can be fully employed in the desired process [170]. All these advantages can be applied to the nanoarchitectonics of nano-/microrobots. From the perspective of nanoarchitectonics on the level of single atoms, 3 different aspects are reviewed in this section: atomic layer deposition (ALD) techniques, point defect engineering, and single-atom decoration.

#### Atomic layer deposition

ALD is an advanced technique for highly controlled fabrication of atomically thin films. During deposition, molecular precursors are coordinated on the desired surface, and in the subsequent step, they are converted into a homogeneous atomic layer. Unlike traditional physical vapor deposition processes, this deposition is driven chemically; therefore, the formed layer is homogeneous and is not affected by substrate nonhomogeneities that can form shadows during traditional physical vapor deposition processes. This method was used by Urso et al. [171] who covered αFe_2_O_3_ peanut-like shaped microrobots with a 30-nm-thick TiO_2_ layer via ALD. The resulting microrobots were propelled in dual modes, that is, they were navigated in a magnetic field and under UV light irradiation. Interestingly, under UV light irradiation, the collective behavior and formation of clusters were dominated owing to the generation of phoretic interactions. In contrast, under dark conditions, the magnetic dipole–dipole interactions oriented the microrobots in parallel. The on–off conditions enabled reversible clustering of microrobots, which was shown to be a suitable solution for the photocatalytic degradation of 2,4-dichlorophenoxyacetic acid, commonly used as an herbicide.

An ALD of metallic layers is also possible. It proceeds via an island growth mechanism (Volmer–Weber mechanism) because of the high surface energy. Exploiting this mechanism, the platinum nanoparticles were grown on tubular microengines that provided the resulting microrobots with the ability of bubble propulsion in the presence of H_2_O_2_ [172]. In this case, 70 deposition cycles of the platinum source were applied. It can be expected that reducing the number of cycles can eventually lead to the formation of single atoms and atomic-level species, as was recently demonstrated for the growth of single palladium atoms [173].

#### Point defect engineering

The presence of defects in the semiconductor structure significantly affects the optical and electrical properties of the resulting material. Defect engineering is traditionally applied in the fabrication of optoelectrical devices, where a precise design of electronic structures is required [174]. Defect-rich structures can also be employed in nanoarchitectonics of nano-/microrobots. Several studies have focused on fabricating nano-/microrobots from defect-rich TiO_2_ that is also known as black titania. Compared to the nondefected TiO_2_ structure, black titania absorbs light in the visible range up to NIR. This is highly convenient as the efficiency of the absorption increases and the entire sun spectrum can be engaged in the desired process. Exploiting this ability, black titania microrobots driven by visible light for biofilm eradication were introduced by Ussia et al. [128]. Alternatively, black titania-based microrobots were described by Lv et al. [175], who described ROS generation under NIR light on a defect-rich structure that exhibited photodynamic effects on tumor inhibition. Although this application was aimed at the medical direction, the generation of ROS that can degrade potential pollutants is also significant for environmental remediation purposes.

**Fig. 8.**
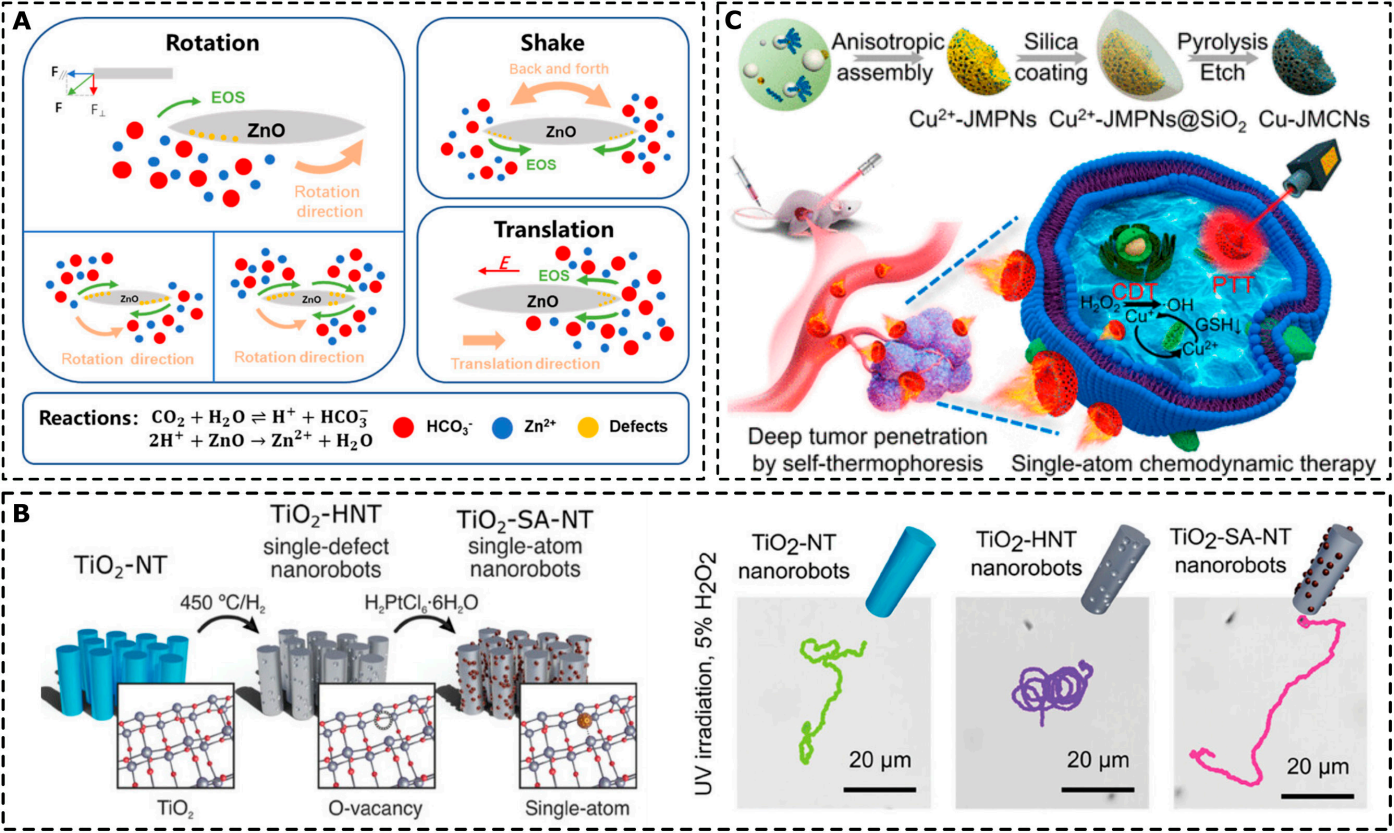
Nanoarchitectonics at the level of single atoms. (A) Influence of the morphology of ZnO-based nanorobots and the distribution of surface defects on propulsion abilities. EOS refers to electro-osmotic slip induced by local diffusion fields of generated ions. Adapted with permission from [176]. Copyright 2023, American Chemical Society. (B) Nanoarchitectonics of TiO_2_ nanotube (NT)-based nanorobots including point defect and platinum single-atom engineering. The right part illustrates different trajectories collected after 20 s in the presence of 5 wt % H_2_O_2_ under UV light irradiation depending on the surface morphology of the nanorobots. Adapted under terms of the CC BY license from [179]. Copyright 2024, Wiley-VCH. (C) Fabrication of N-doped jellyfish-like mesoporous carbon nanorobots decorated with Cu single atoms (Cu-JMCNs) and the illustration of the self-thermophoresis-driven propulsion abilities. Adapted with permission from [180]. Copyright 2023, American Chemical Society.

The effect of surface defects in light-driven microrobots on the resulting propulsion abilities can be generalized by defect engineering in semiconductors. Liu et al. [176] conducted a comprehensive study on ZnO nanoshuttle-like microrobots. As demonstrated, the homogeneity of the surface defects can predetermine the preferred mode of propulsion due to the formation of nonhomogeneous ionic species over the body of the nanorobots (Fig. 8A). One-sided defects or different densities of defects caused preferable rotation of nanorobots, roughly symmetrical surface defects induced shaking-like motion, and, finally, surface defects dominating only on the axial side led to the translation behavior.

#### Single-atom decoration

Fine decoration of nano-/microrobots with single atoms has been demonstrated for 2 main purposes: providing or adjusting propulsion abilities and providing the resulting nano-/microrobots with catalytic abilities [177]. Regarding the propulsion of nano-/microrobots, single iron atoms were implemented on the surface of hierarchically porous graphitic carbon, and they catalytically decomposed H_2_O_2_ fuel, leading to bubble-propelled motion [178]. On the contrary, single platinum atoms implemented on the surface of TiO_2_-based nanorobots were not sufficient for bubble propulsion by H_2_O_2_ decomposition (Fig. 8B). However, in synergy with the TiO_2_ structure and surface defects, the nanorobots were propelled by ionic diffusiophoresis in the presence of H_2_O_2_ and under UV irradiation. In the case of platinum single-atom decorated nanorobots, negative photogravitaxis was observed. The presence of platinum single atoms and atomic-level species was described not only to influence the resulting trajectory of the nanorobots but also the affinity for nanoplastic capture [179].

Alternatively, the generation of ROS over single atoms can be used toward degradation purposes. The generation of ROS over single copper atoms dispersed on jellyfish-like mesoporous carbon nanomotors powered by self-thermophoresis under NIR light irradiation was demonstrated by Xing et al. [180]. In their study, nanorobots were used as candidates for nanocatalytic therapy in bioapplications (Fig. 8C). Similarly, ROS generation was facilitated by single iron atoms that were implemented on graphitic nanorobots decorated with magnetic nanoparticles [181]. In this design, single iron atoms were responsible for ROS generation under acidic conditions that were used for *Helicobacter pylori* eradication in the human stomach.

To conclude, the nanoarchitectonics on the level of single atoms provides a solution for tuning propulsion abilities, electronic properties, or catalytic properties with high precision while eliminating the resources and the materials used and enabling the availability of the main body of nano-/microrobots for additional interactions. The presence of single atoms or point defect engineering seems to be a perspective solution for precise navigation of nano-/microrobots in desired environment. Furthermore, it clearly affects the interaction with targeted pollutants, such as microplastics, and eventually offers photocatalytic abilities toward elimination of resistant bacterial species.

## Outlook and Conclusion

**Fig. 9.**
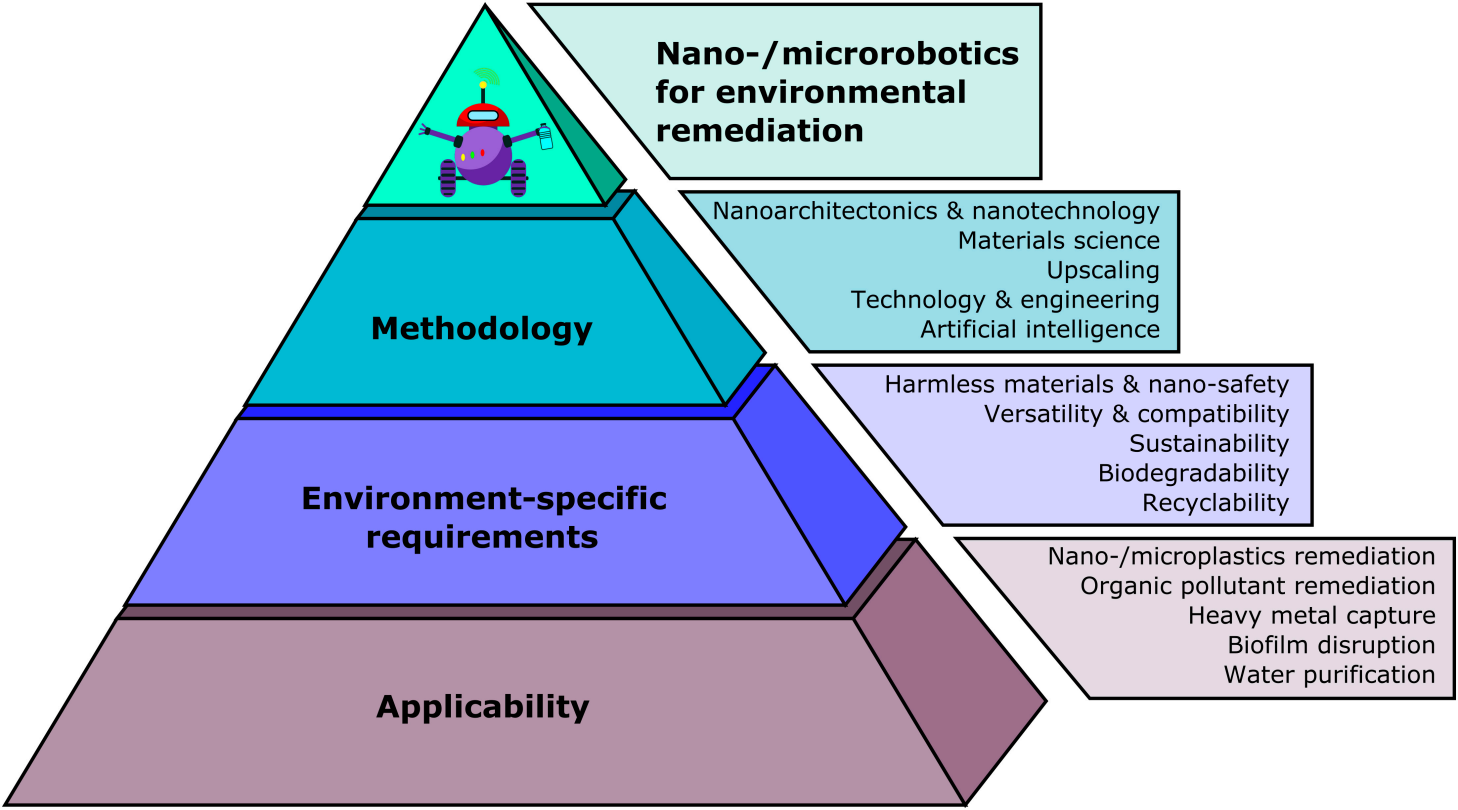
A scheme illustrating application of nano-/microrobots for environmental remediation suggesting different aspects in future real-scale directions.

The application of nano-/microrobots toward environmental remediation seems to be highly advantageous because of the extraordinary efficiency given by the active character arising from propulsion abilities combined with the great versatility of the functionality. The versatility is intensified by applying the general rules of nanoarchitectonics to the engineering of nano-/microrobots. However, research on nano-/microrobots for environmental remediation is still in the stage of a basic research, and one should be aware of the obstacles that need to be overcome. Figure 9 suggests different stages of the application of nano-/microrobots including several selected aspects. The application demands in various environments are quite clear, i.e., the need to focus on nano-/microplastics and organic pollutant remediation, heavy metal detection and capture, biofilm elimination, and overall water purification. Similarly, the specifics of each environment can also be approached by carefully designing nano-/microrobots to meet safety requirements and sustainability while ensuring the compatibility. At the current stage of the development, the methodology stage is currently scrutinized from different aspects, such as nanoarchitectonics and nanotechnology to enable efficient design and visualization, materials science to maintain the sustainability and scalability while keeping the maximal efficiency, technology and engineering to scale the processes into industrially sustainable solutions, and lastly artificial intelligence to maximize the overall efficiency.

One of the main concerns of the application of nano-/microrobots is the potential danger of using nano- and microparticles [182–184]. Currently, the application of nano-/microrobots openly in groundwater or oceans is not very likely because they still must be treated as nano-/micromaterials that represent a potential risk of additional pollution in the environment. To pursue the application of nano-/microrobots for a direct treatment in open environments, more attention to the choice of the material and its compatibility with the system must be paid. Simultaneously, the impact of the nanoscaled morphology must be considered as the dimensionality influences the toxicity of materials. However, nano-/microrobots can be of significant help when used in controlled and closed environments such as sewage treatment plants. Taking into account the dynamic character of nano-/microrobots that enable action in the “on-the-fly” mode, great efficiency in remediation processes can be expected. A different possible path toward pollutant remediation at this stage could be in living systems. The use of nano-/microrobots for antibiotic-resistant bacterial infections seems to be highly perspective. Similarly, nano-/microrobots could be of a great help in detection and monitoring of the nano-/microplastics in living organisms.

The next possible limitation of a large-scale application arises from the complexity of the nano-/microrobots. The powerful remediation abilities are achieved by precise nanoengineering: The nano-/microrobots can typically provide a solution for a specific contaminant treatment. Considering this aspect, the design and functionality of nano-/microrobots should be as universal as possible to deal with a broad variety of contaminants [23]. At this stage, the materials science is supposed to play a crucial role to design suitable advanced materials; depending on the targeted environment, a general type of the pollutant can be modeled and approached. For example, suitable surface charge as well as an appropriate choice of photocatalytic material can enable fabrication of nano-/microrobots suitable for photodegradation of various microplastics ranging from polylactic acid (PLA), PCL, polyethylene terephthalate (PET), and polypropylene (PP) [137].

Finally, pursuing with the real-scale environmental remediation with the assistance of nano-/microrobots still requires sustainable, compatible, and cost-effective industrial solutions. There are already some promising ideas on how to transfer nano-/microrobots into operational large-scale environment remediation systems, such as designing closed water-remediation flow systems that can provide efficient water treatment due to the precisely designed setup providing the ability to propel nano-microrobots in different modes while ensuring the safety of the process [30]. In the next step, tight cooperation with the industry and engineering is necessary to test and optimize the setups and related technologies.

In a broader perspective, it is important to consider the implications of this review. The present review primarily concerns the application of environmental purification with nano-/microrobots. However, the postulated methodologies are also relevant in other fields. A variety of approaches are being taken in the field of material science to address the challenges currently facing humanity. These include the development of materials that contribute to energy problems [185–187], sensing materials that detect the environment and biological risks [188–190], materials that are useful in biomedicine [191–193], and materials that can be used in devices for information handling [194–196]. From this regard, nanoarchitectonics represents the methodology to efficiently organize these materials into functional autonomous systems applicable beyond the environmental applications. We can say that the nanoarchitectonics of nano-/microrobots provides a reliable passport for the development of intelligent advanced functional next-generation systems across a range of disciplines: expanding not limitedly into bioapplications, medical applications, energy conversion, food industry, chemical industry, and agriculture.

To conclude, the presented review summarized the concepts of nanoarchitectonics projected in the field of nano-/microrobots for environmental applications. With the tools of nanoarchitectonics, advanced functional nano-/microrobots can be engineered toward efficient environmental remediation. The aim of the review was to discuss the general approaches of nanoarchitectonics toward the fabrication of advanced smart nano-/microrobots. Simultaneously, general concepts were provided to help to assess the potential synergy between morphology, materials, functionalization, and interactions toward efficient environmental remediation. By engineering nano-/microrobots with the eyes of nanoarchitectonics, effective solutions for environmental remediation can be designed, ranging from highly selective pollutant treatment to more universal solutions for complex operations and beyond.
